# Olfactory Plasticity: Variation in the Expression of Chemosensory Receptors in *Bactrocera dorsalis* in Different Physiological States

**DOI:** 10.3389/fphys.2017.00672

**Published:** 2017-09-14

**Authors:** Sha Jin, Xiaofan Zhou, Feng Gu, Guohua Zhong, Xin Yi

**Affiliations:** ^1^Key Laboratory of Crop Integrated Pest Management in South China, Ministry of Agriculture, South China Agricultural University Guangzhou, China; ^2^Key Laboratory of Natural Pesticide and Chemical Biology, Ministry of Education, South China Agricultural University Guangzhou, China; ^3^Guangdong Province Key Laboratory of Microbial Signals and Disease Control, Integrative Microbiology Research Centre, College of Agriculture Guangzhou, China

**Keywords:** olfactory plasticity, odorant receptor, ionotropic receptor, gustatory receptor, *Bactrocera dorsalis*

## Abstract

Changes in physiological conditions could influence the perception of external odors, which is important for the reproduction and survival of insect. With the alteration of physiological conditions, such as, age, feeding state, circadian rhythm, and mating status, insect can modulate their olfactory systems accordingly. Ionotropic, gustatory, and odorant receptors (IR, GR, and ORs) are important elements of the insect chemosensory system, which enable insects to detect various external stimuli. In this study, we investigated the changes in these receptors at the mRNA level in *Bactrocera dorsalis* in different physiological states. We performed transcriptome analysis to identify chemosensory receptors: 21 IRs, 12 GRs, and 43 ORs were identified from *B. dorsalis* antennae, including almost all previously known chemoreceptors in *B. dorsalis* and a few more. Quantitative real-time polymerase chain reaction analysis revealed the effects of feeding state, mating status and time of day on the expression of IR, GR, and OR genes. The results showed that expression of chemosensory receptors changed in response to different physiological states, and these changes were completely different for different types of receptors and between male and female flies. Our study suggests that the expressions of chemosensory receptors change to adapt to different physiological states, which may indicate the significant role of these receptors in such physiological processes.

## Introduction

Insects need to respond to environmental cues in line with their own physiological states. Olfactory plasticity enables them to modify their response to odors according to age, feeding state, circadian rhythm, or mating status (Gadenne et al., 2016). For example, a sense of satiety partly determines food ingestion behaviors (Croll and Chase, 1980). Starvation can increase the response of *Drosophila melanogaster* to food signals, the effect escalates over the starvation time (Edgecomb et al., 1994). In mosquitoes, a blood meal can promote ovarian development and inhibit feeding behaviors (Klowden and Lea, 1979a,b). After ingesting sufficient amounts of blood, mosquitoes devote more energy to find suitable oviposition sites and become more attracted to relevant signals (Gadenne et al., 2016). The regulation of olfactory system also occurred upon mating at all levels. Generally, after mating, insects become less sensitive to sexual signals, whereas are inclined to be attracted by oviposition-site cues or food odors. The antennal neurons become less sensitive to sex pheromones, while the responses to host plant odors are unchanged after mating in *Spodoptera littoralis* males (Kromann et al., 2015). Moreover, like many other activities, olfactory behaviors vary by the time of day (Gadenne et al., 2016). Studies showed that olfactory sensory neurons and antennal sensitivity of many insect species tend to be controlled by the biological clock or body rhythm (Krishnan et al., 1999; Page and Koelling, 2003).

Three molecular components of the insect chemosensory system have been identified to be significant for the perception and recognition of odorant stimuli: the odorant receptors (ORs), the ionotropic receptors (IRs), and gustatory receptors (GRs) (Benton et al., 2009; Agnihotri et al., 2016; Shen, 2017). They are indispensable for detecting a wide range of environmental stimuli: bitterness, sweetness, odor, pheromones, humidity, carbon dioxide, and carbonated water (Bargmann, 2006; Vosshall and Stocker, 2007). To cope with various external stimuli, olfactory plasticity may regulate insect olfactory system by changing the expressions of these receptors when the insect enters specific physiological states (Su and Wang, 2014).

The oriental fruit fly, *Bactrocera dorsalis* (Hendel), is one of the main fruit pests in the Asia-Pacific region. They inhabit broad range of host species, have wide climate tolerance, display high dispersal capacity, and significant fecundity (Hsu et al., 2016). During development, adult oriental fruit fly lay eggs inside the fruits of different types of host plants for feeding and oviposition (Zheng et al., 2012). This makes the fly a highly invasive polyphagous species. Due to the direct damage to crops and negative effects on export markets, they are considered a quarantine pest. Our group has conducted a series of studies concerning the chemosensory system of oriental fruit fly (Yi et al., 2013, 2014). In *B. dorsalis*, chemosensory perception plays a key role in behavior regulation such as, host-seeking, mating and oviposition. It also exhibits remarkable developmental phases in olfactory behaviors (Wu et al., 2016). In this study, we examined the *de novo* transcriptome of *B. dorsalis* and identified 21 IR, 12 GR, and 43 OR genes. To test whether insect can modulate their olfactory system via the changes in the expression of chemosensory receptors according to their physiological states, we performed quantitative real time PCR to examine expression patterns of receptor genes to different physiological states, including feeding states, time of day and mating status. Our results illuminate the potential role of the chemosensory receptors in physiological state-dependent forms of olfactory plasticity.

## Materials and methods

### Insect rearing

*B. dorsalis* were obtained from a laboratory-reared stock colony (Key Laboratory of Pesticide and Chemical Biology, South China Agricultural University, Guangzhou, China) and maintained at 28°C in 70% relative humidity, with a 14:10 (light: dark) photoperiod. Adult flies were reared on artificial diets consisting of yeast extract, sugar, honey, and agar (Wu et al., 2015).

### RNA isolation

Total RNA was isolated from antennae from female and male adult flies (female/male ratio = 1:1) at different stages, including 2 d after eclosion, sexual immaturity (8 d after eclosion), sexual maturity but unmated (12 d after eclosion, females and males in two separate cages before maturity), and mated (15 d after eclosion). Antennae isolated from flies of different stages were mixed together, and the amount of the antennae sample was more than 5 mg. We extracted total RNA by the RNA isolation kit (Omega, USA) according to the manufacturer's instructions. We measured the concentration of isolated RNA using Nanodrop (Thermo Fisher Scientific, USA).

### Construction of the cDNA library and illumina sequencing

We constructed the cDNA library using an Illumina kit following the manufacturer's recommendations. mRNA was split into small fragments after purification with oligo (dT) magnetic beads. Using mRNA as a template, we synthesized the first strand of cDNA using a random hexamer primer. Then, to obtain double-strand cDNA, we added buffer for reverse transcriptase, dNTPs, RNase H, and DNA polymerase I. For end repair and poly (A) addition, the double-stranded cDNA was purified. Finally, the 5′ and 3′ ends of the fragments were ligated. Suitable fragments, as judged by agarose gel electrophoresis, were selected for use as templates for PCR amplification to create a cDNA library. The cDNA library was sequenced on an Illumina sequencing platform (HiSeqTM 2000) and 100 bp paired-end reads were generated (Zhang et al., 2015).

### Identification of olfactory genes

Trinity was used to perform *de novo* transcriptome assembly on the filtered reads. We further processed the initial assembly generated by Trinity by CD-HIT to remove redundant transcripts and by COREST to cluster together transcripts that shared a high number of reads. We identified potential gene coding regions (hereafter “genes”) from the final transcriptome assembly using TransDecoder. Then we analyzed translated amino acid sequences of all identified genes using InterProScan v5.23 and the sequences that contain the characteristic domains of insect chemosensory receptors (IR: IPR001320; GR: IPR009318 and IPR013604 and OR: IPR004117) were identified as *B. dorsalis* IR, GR, and OR proteins, respectively.

For each of the IR, GR, and OR gene families, we aligned amino acid sequences of genes in *B. dorsalis* (identified in this study) and *D. melanogaster* (collected from previous studies) using MAFFT v7.310 with the high accuracy option “E-INS-I.” Columns with high proportion of gaps were filtered from the resultant multiple sequence alignments using TrimAL v1.4 with the “-gappyout” option. Evolutionary models best fitting the data were selected using the “ModelFinder” feature of IQ-TREE v1.5.4, the models “LG+F+R5” and “LG+F+R7” were selected for OR/GR and IR, respectively. Phylogenetic reconstructions were then conducted on the filtered alignments using IQ-TREE v1.5.4 with the “-nstop” parameter (maximum number of continuous unsuccessful iterations) set to 500. The reliabilities of estimated phylogenetic trees were assessed using the ultra-fast bootstrap (1,000 replicates) implemented in IQ-TREE v1.5.4. The phylogenetic trees were visualized using the interactive tree of life (iTOL) server v3.

### Collection of sample in different physiological states

For the assessment of feeding state, flies (15 d after eclosion) were starved for 4 h, or 8 h, respectively; un-starved flies were kept in cages with the usual food supply. There were 100 flies (female or male) in each experimental group, and each treatment was performed with three replicates. After starvation, antennae from female and male flies were isolated separately. The antennae were collected at the same time and stored at −80°C before RNA extraction.

For the assessment of mating states, sexually mature female and male flies (14 d after eclosion) were separated into two different cages to avoid mating, but were raised under the same conditions. Female and male flies in the mated group were raised together in the same cage. There were 100 flies (female or male) in each experimental group, and each treatment was performed with three replicates. The antennae of all groups were collected at the same time and stored at −80°C before RNA extraction. Female and male flies were examined separately.

Gravid female flies were collected at different times of day (9 a.m., 4 p.m., and 10 p.m.) to test olfactory gene expression levels at different time points. Only female flies were included in each group with three replicates. The antennae were collected at the same time and were stored at −80°C before RNA extraction.

### Expression level examination

We investigated expression patterns of all identified olfactory genes by qRT-PCR. qRT-PCR was performed as previously reported (Yi et al., 2014). We reverse-transcribed 1 μg isolated RNA to first-strand cDNA by using M-MLV reverse transcriptase (TaKaRa, China) and oligo(dT)_18_ as primer at 42°C for 60 min. The reaction was terminated by heating at 95°C for 5 min, and the products were stored at −20°C. We performed qRT-PCR using the iCycler iQ Real-Time PCR Detection System (Bio-Rad, Hercules, CA, USA) with SYBR green dye (Taraka, China) binding to double-strand DNA at the end of each elongation cycle. Amplification was performed by the primers listed in Supplementary Table 1. All amplifications were performed with three biological replicates. We analyzed relative gene expression data using the 2-ΔΔCT method as described by Livak and Schmittgen (2001).

### Heat mapping

Phylogenetic trees were made with the maximum likelihood method with multiple alignments of amino acid sequences of identified BdorIRs, BdorGR, and BdorORs, respectively. Bootstrapping supports were indicated beside the branches at 1,000 simulations. Changes at expression levels were calculated as the ratio of different experimental groups. Log_2_ scale of fold changes of each treatment group relative to the control group were shown in the heat map. Orange color indicated that the expression level was significantly increased, while blue color indicated that the expression level was significantly decreased. White color indicated that the expression levels between two different treatments were not significant changed.

### Statistical analysis

All results from experimental replicates were expressed as means (±S.E.M) and analyzed with one-way analysis of variance or *t*-tests using SPSS 17.0 for Windows (SPSS Inc., Chicago, IL, USA).

## Results

### Identification of *B. dorsalis* chemosensory receptors by *De novo* transcriptome assembly

**Figure 1:**
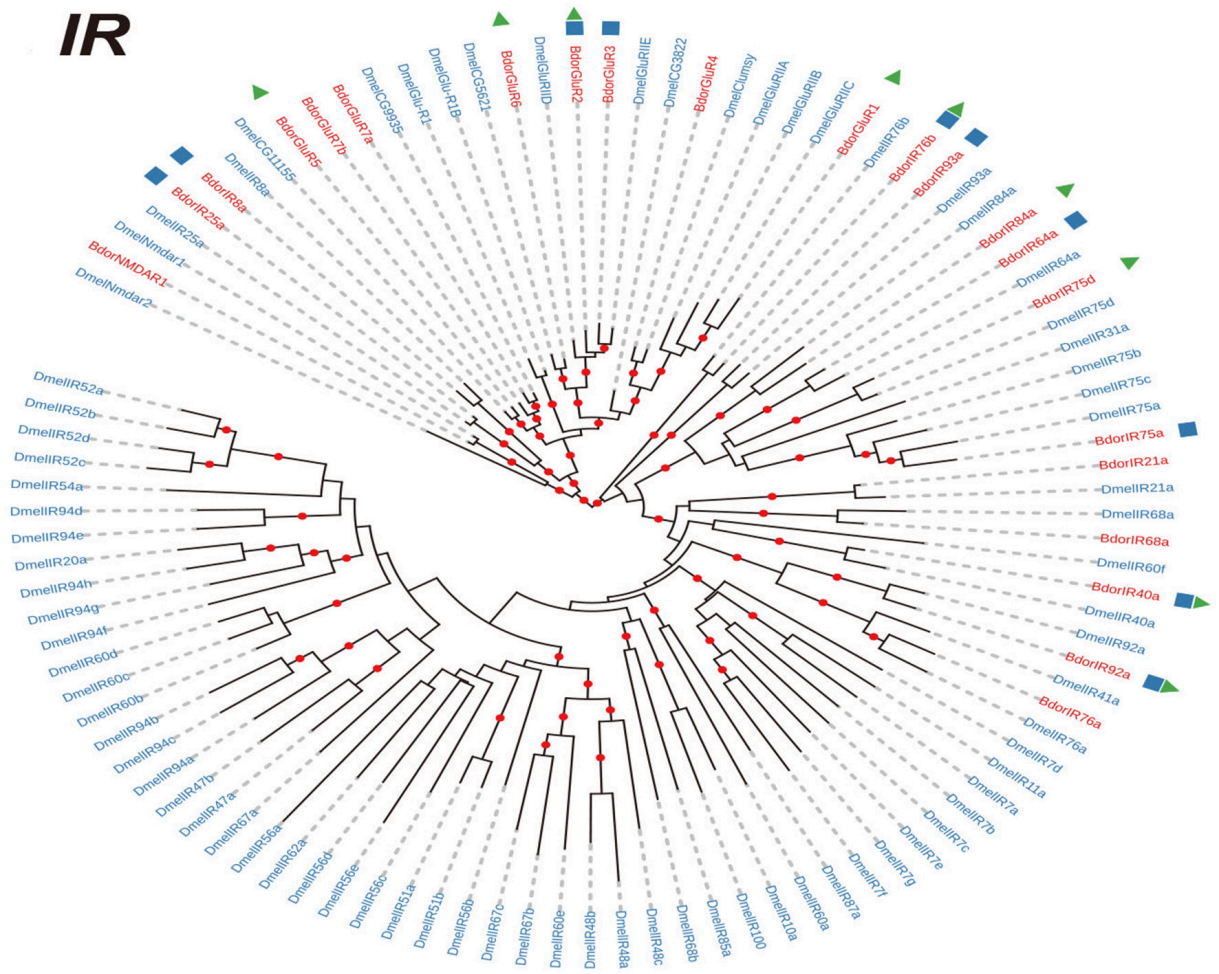
Phylogenetic trees of IR genes. Phylogenetic trees were constructed with the maximum likelihood method. Phylogenetic relationships of IR genes in *Bactrocera dorsalis* and *Drosophila melanogaster*. The genes of *B. dorsalis* and *D. melanogaster* are highlighted in red and blue, respectively. The blue cube indicates the receptors identified in Liu et al. (2016), and the red triangle indicates the receptors identified in Wu et al. (2015). IR, ionotropic receptor.

**Figure 2:**
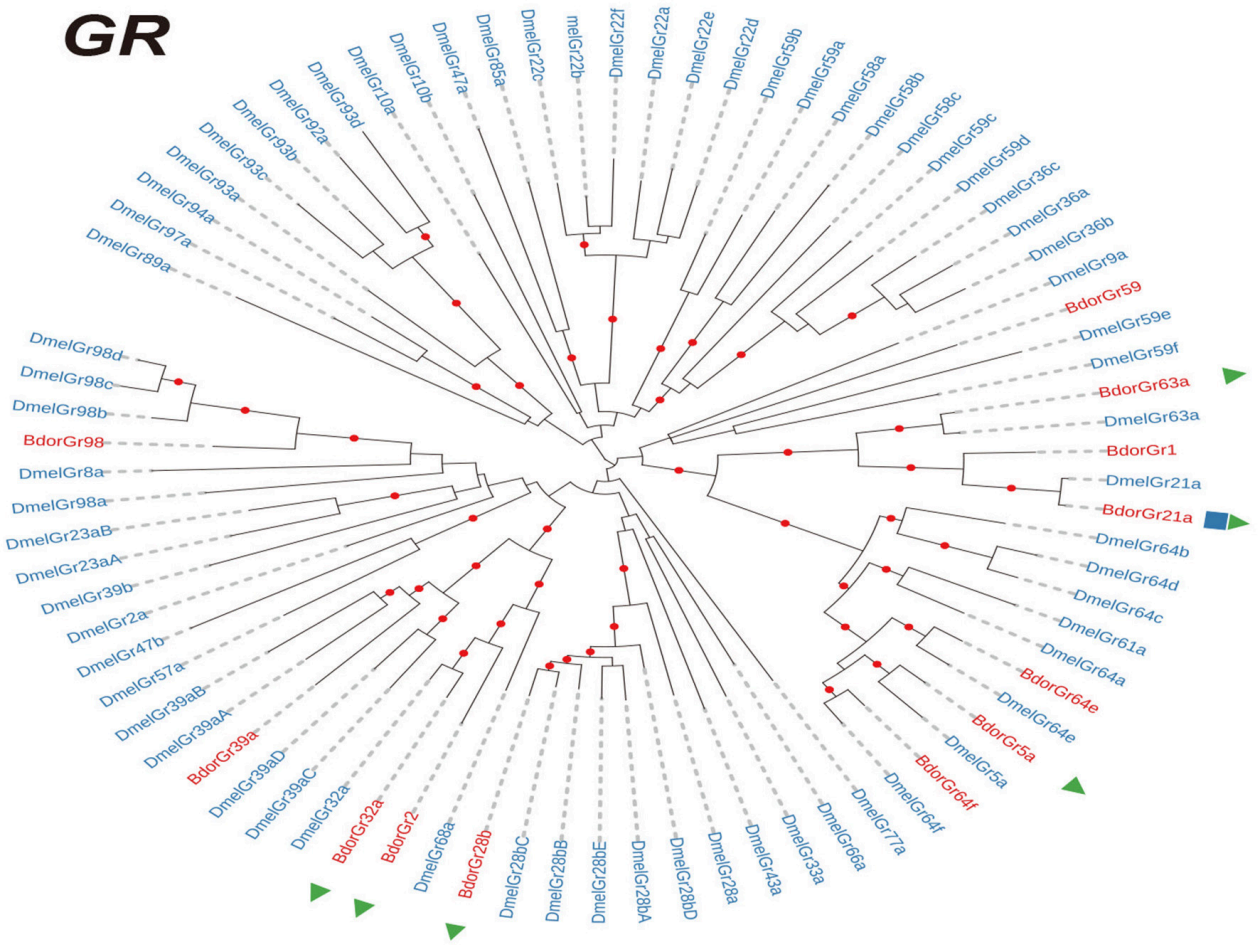
Phylogenetic trees of GR genes. Phylogenetic trees were constructed with the maximum likelihood method. Phylogenetic relationships of GR genes in *Bactrocera dorsalis* and *Drosophila melanogaster*. The genes of *B. dorsalis* and *D. melanogaster* are highlighted in red and blue, respectively. The blue cube indicates the receptors identified in Liu et al. (2016), and the red triangle indicates the receptors identified in Wu et al. (2015). GR, gustatory receptor.

**Figure 3:**
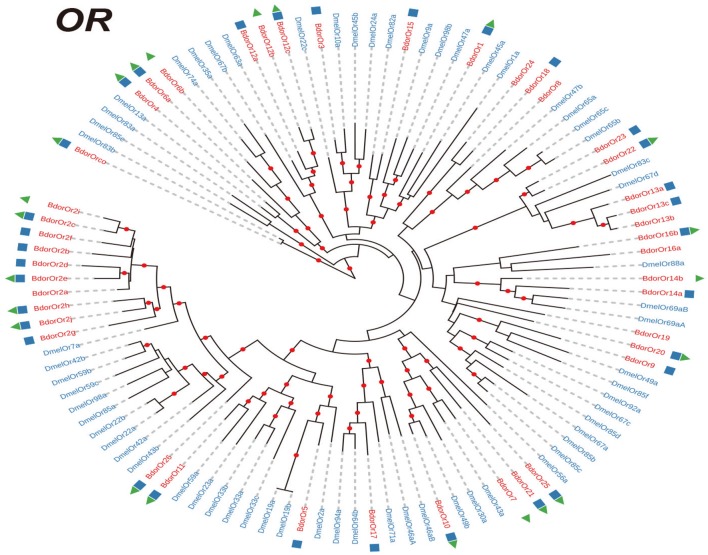
Phylogenetic trees of OR genes. Phylogenetic trees were constructed with the maximum likelihood method. Phylogenetic relationships of OR genes in *Bactrocera dorsalis* and *Drosophila melanogaster*. The genes of *B. dorsalis* and *D. melanogaster* are highlighted in red and blue, respectively. The blue cube indicates the receptors identified in Liu et al. (2016), and the red triangle indicates the receptors identified in Wu et al. (2015). OR, odorant receptor.

In total, 25,232,772 clean reads were obtained from the antennal transcriptome of *B. dorsalis*. These reads were assembled into 56,899 unigenes, with an average length of 1449.02 bp and an N50 of 2131. 93.4 % clean reads aligned on the assembly. The data were deposited at National Center for Biotechnology Information (NCBI) under SAR database, with the accession number SRR5801940. We then performed the *de novo* assembly of the antennal transcriptome of *B. dorsalis* and identified 21 IRs, 12 GRs genes, and 43 ORs (Supplementary Datasheet 1). Then we investigated the evolutionary relationships among the IR, GR, and OR genes in *B. dorsalis* and *D. melanogaster*. Our results showed that most *B. dorsalis* chemoreceptor genes (19/21 IRs, 9/12 GRs, and 30/43 ORs) had well-supported (co-)orthologs in *D. melanogaster* (Figures 1–3). As shown in Figures 1, 2, the IR and GR genes in *B. dorsalis* were named after their counterparts in *D. melanogaster* when our phylogenetic analyses indicated clear orthologous relationships between them. For OR genes, however, evolutionary relationships between *B. dorsalis* and *D. melanogaster* genes were much more complicated: most *B. dorsalis* OR genes had either no or multiple orthologous genes in *D. melanogaster*. Therefore, for this gene family, we chose to rename *B. dorsalis* genes in numerical order (Figure 3). Moreover, many *B. dorsalis* chemoreceptor genes exhibited one-to-one relationship with their counterparts in *D. melanogaster*, including the highly conserved *Orco* (Or co-receptor) and “antennal” IR genes. However, a few species-specific gene duplications were also observed. Note that there was a *B. dorsalis-*specific clade in the OR family consisting of 10 genes, while the ortholog in *D. melanogaster* (*DmelOr7a*) contain single-copy (Figure 3), which suggested that *B. dorsalis* expanded rapidly after the divergence of the ancestors of the two species. We also compared the sequences identified in this study with those of previous studies (Supplementary Table 2). Our study covered almost all receptors reported in previous papers: 10/11 IRs, 6/6 GRs, and 22/23 ORs in study of Wu et al. (2015) and 10/12 IRs, 32/35 ORs in study of Liu et al. (2016), indicating a very robust analysis of our data.

### Variations in chemosensory receptor expression before and after mating

**Figure 4:**
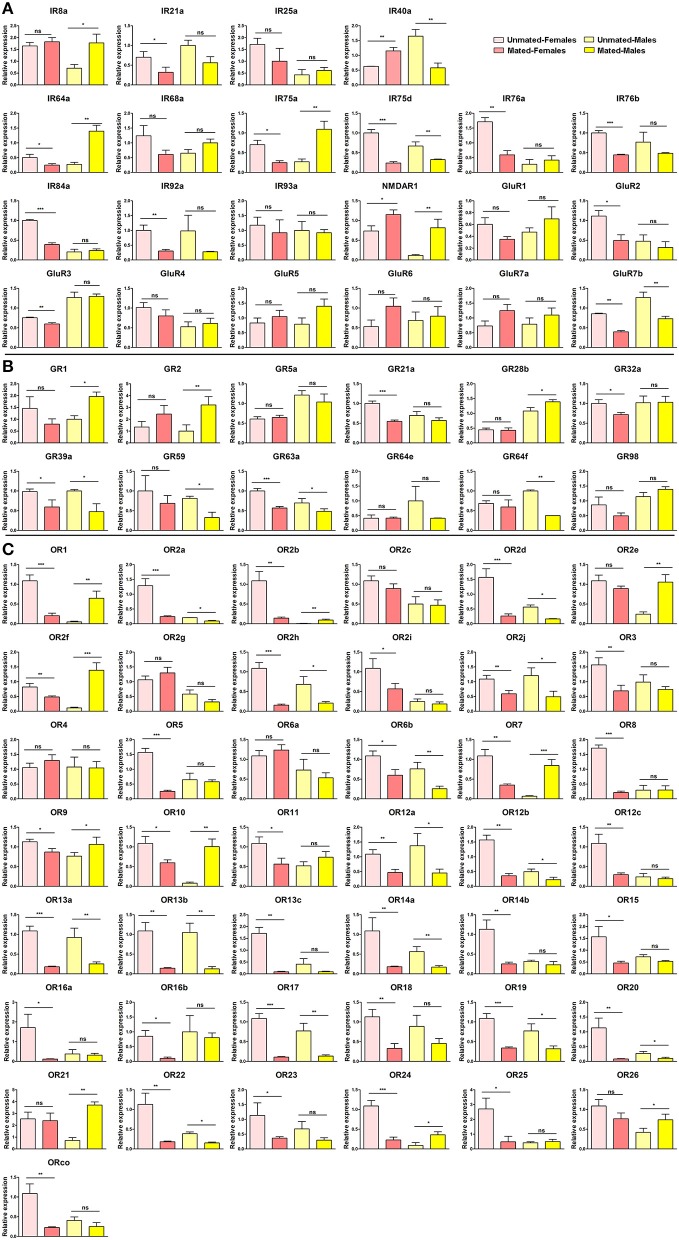
Variation in chemosensory receptors in *Bactrocera dorsalis* before and after mating. Variations in **(A)** BdorIRs, **(B)** BdorGRs, and **(C)** BdorORs expression in *B. dorsalis* before and after mating. The data represent the mean ± S.E.M of three replicates (^***^p < 0.001, ^**^p < 0.01, ^*^p < 0.05, *t*-test).

To reveal how mating affects the expression of chemosensory receptors in *B. dorsalis*, we measured expression levels of the receptors before and after mating by qRT-PCR. In total, 13 IRs in females and 7 IRs in males showed significant changes after mating. However, the pattern of change was different between male and female. Expression of most IRs (16/21) in female flies was down-regulated after mating. In contrast, expression of most IRs (13/21) in male flies was increased after mating (Figure 4A). Most GRs showed a negligible change in expression after mating. However, a few GRs showed similar changes in males and females (the expression of GR2 and GR28b increased in both male and female; and the expression of GR21a, GR39a, GR59, GR63a, GR64f decreased in both male and female). The largest changes occurred in GR21a of female flies and GR2 of male flies, which decreased 1.83-fold and increased 3.20-fold, respectively, after mating (Figure 4B), which indicates their potential role in mating or after mating. The expression levels of ORs in *B. dorsalis* varied significantly after mating. Almost all identified ORs (39/43) were down-regulated by mating in female flies (Figure 4C). However, the pattern of changes in male flies had distinct differences from that in female (10 ORs were up-regulated and 14 ORs were down-regulated by mating). This indicates that ORs may function differentially in sensing male and female pheromone during mating processes. Specifically, the expression of OR10 and OR2f in mated males was 12.41-fold and 17.11-fold higher than that in unmated male flies, implying an essential requirement of these two ORs in sexual pheromone sensing in male flies (Figure 4C).

### Variation in chemosensory receptor expression at different points during egg laying

**Figure 5:**
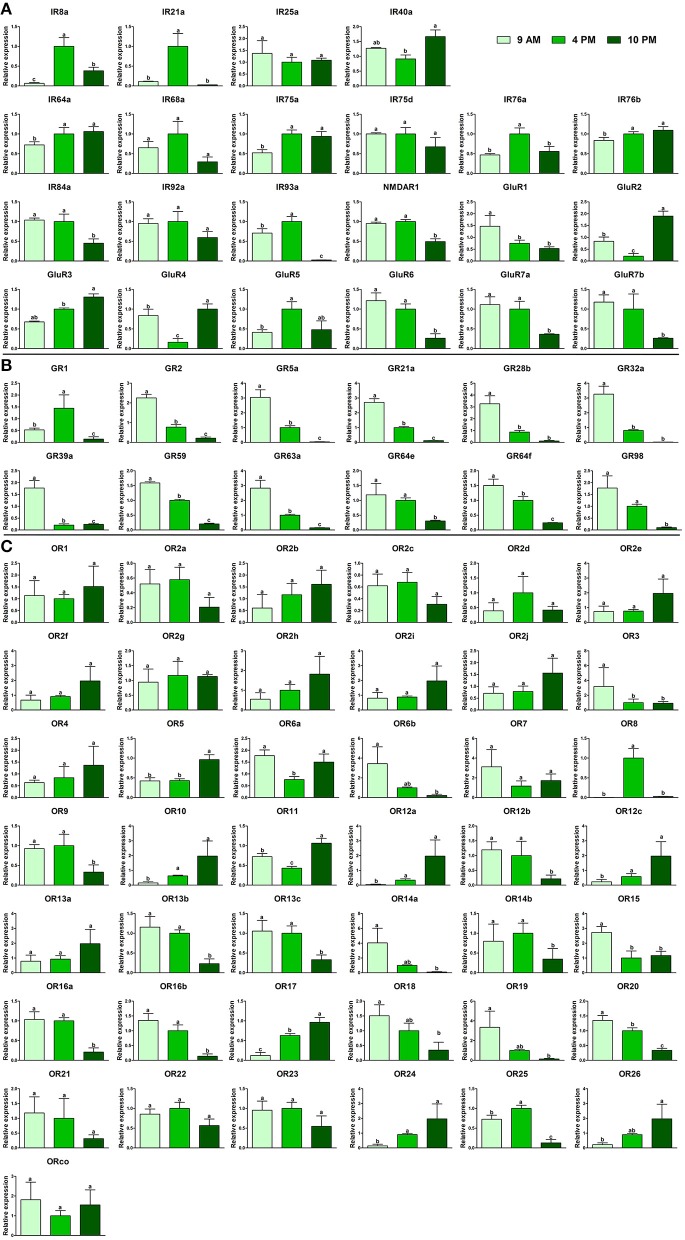
Variation in chemosensory receptors at different time of the day in female *Bactrocera dorsalis* during the egg-laying phase. Variations in **(A)** BdorIRs, **(B)** BdorGRs, and **(C)** BdorORs expression at different times of day in female *B. dorsalis*. The data represent the mean ± S.E.M of three replicates. Different letters indicate significant difference in the expression (*p* < 0.05, one-way analysis of variance).

Next we studied expression at different times of the day. Because the egg—laying peak of *B. dorsalis* occurred at 4 p.m., we collected flies at 9 a.m., 4 p.m. and 10 p.m. to analyze the expression levels of the chemosensory receptors before and after the egg laying peak. For all IRs, expression at 9 a.m. and 4 p.m. tended to be stable, and the highest or lowest expression often occurred at 10 p.m. Six IRs (IR40a, IR64a, IR76b, GluR2, GluR3 and GluR4) showed the highest expression at 10 p.m., while 11 IRs showed the lowest expression at this time. The expression of five IRs (IR8a, IR21a, GluR2, GluR4, and GluR5) displayed sharp fluctuations over these three time points (Figure 5A). Except for GR1, all other GRs showed downtrend of expression along the three time points, reaching their lowest point at 10 p.m. (Figure 5B). Similar to IRs, many ORs (17/43) exhibited the highest expression at 10 p.m., whereas 19 ORs showed the lowest expression at this time (Figure 5C). In addition, 15 ORs showed the highest expression at 9 a.m., whereas 11 ORs showed the highest expression levels at 4 p.m., which indicated that these ORs may be involved in oviposition behaviors. The expression levels of 15 ORs showed tendency to descend along with the timeline, while the expression levels of 12 ORs were on the decrease along with the time, which indicated that the expression changes of these ORs may follow an internal clock to maintain a certain rhythm.

### Variation in chemosensory receptor expression in different feeding conditions

**Figure 6:**
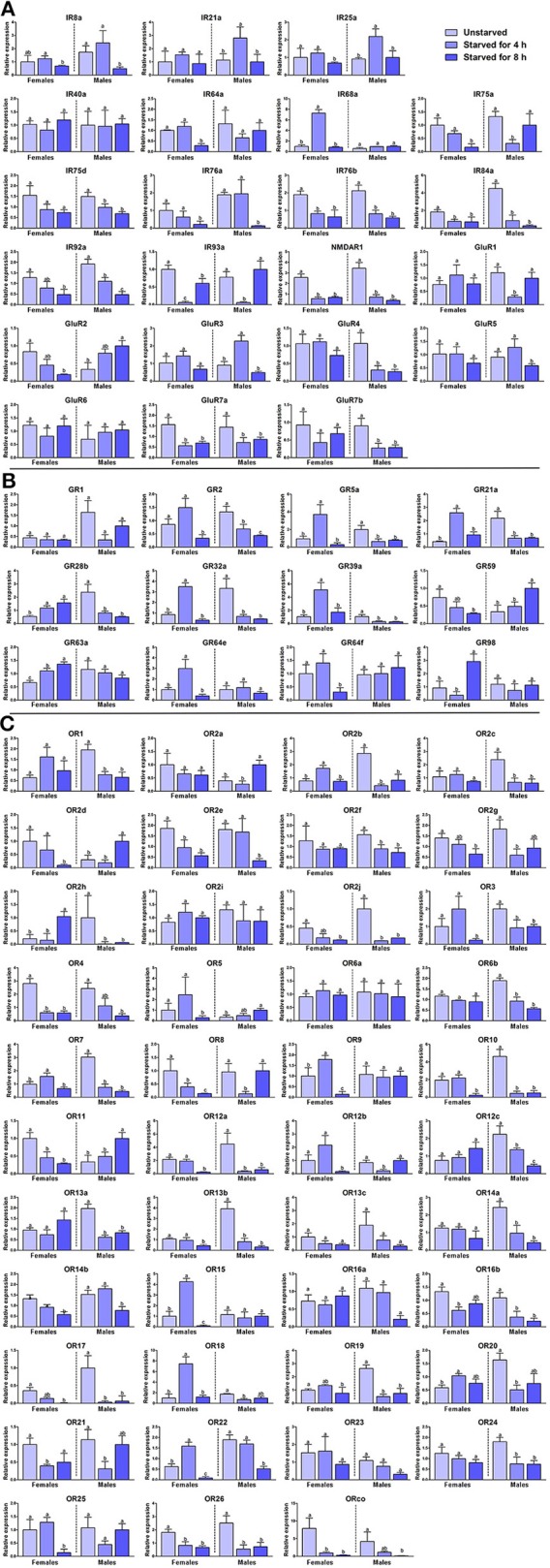
Variation in chemosensory receptors at different feeding states in *Bactrocera dorsalis*. Variation in **(A)** BdorIRs, **(B)** BdorGRs, and **(C)** BdorORs expression at different feeding states of *B. dorsalis*. The data represent the mean ± S.E.M of three replicates. Different letters indicate significant differences in expression (*p* < 0.05, one-way analysis of variance).

Feeding state also affects the chemosensory recognition behaviors of insects. Flies were starved for 4 or 8 h (the survival rate was 100% after starvation treatment), and expression of the receptors was measured with un-starved flies serving as the control. Starvation induced decreased expressions of most IRs in both males and females, with only a few exceptions that showed dramatic increase after 4 h starvation (IR21, IR68a, IR93a in female flies, and GluR3, IR25 in male flies; Figure 6A). For GRs, 4 h of starvation induced rapid increase of expression of seven GRs in female flies, followed by decrease with the prolongation of starvation time. In male flies, starvation brought about a sustained decrease in the expression of most GRs (Figure 6B). Expression of most ORs decreased after starvation in both female and male flies. Some exceptions occurred at 4 h starvation time point in female flies: some ORs showed increased expression after 4 h starvation. This may indicate that female flies become more sensitive to food odors after 4 h of starvation, and those ORs may play a role in detecting food chemicals. Three ORs in male flies were up-regulated by starvation. The increased expression of these three genes may be involved in food-searching behavior in starved male flies (Figure 6C).

### Heat mapping

**Figure 7:**
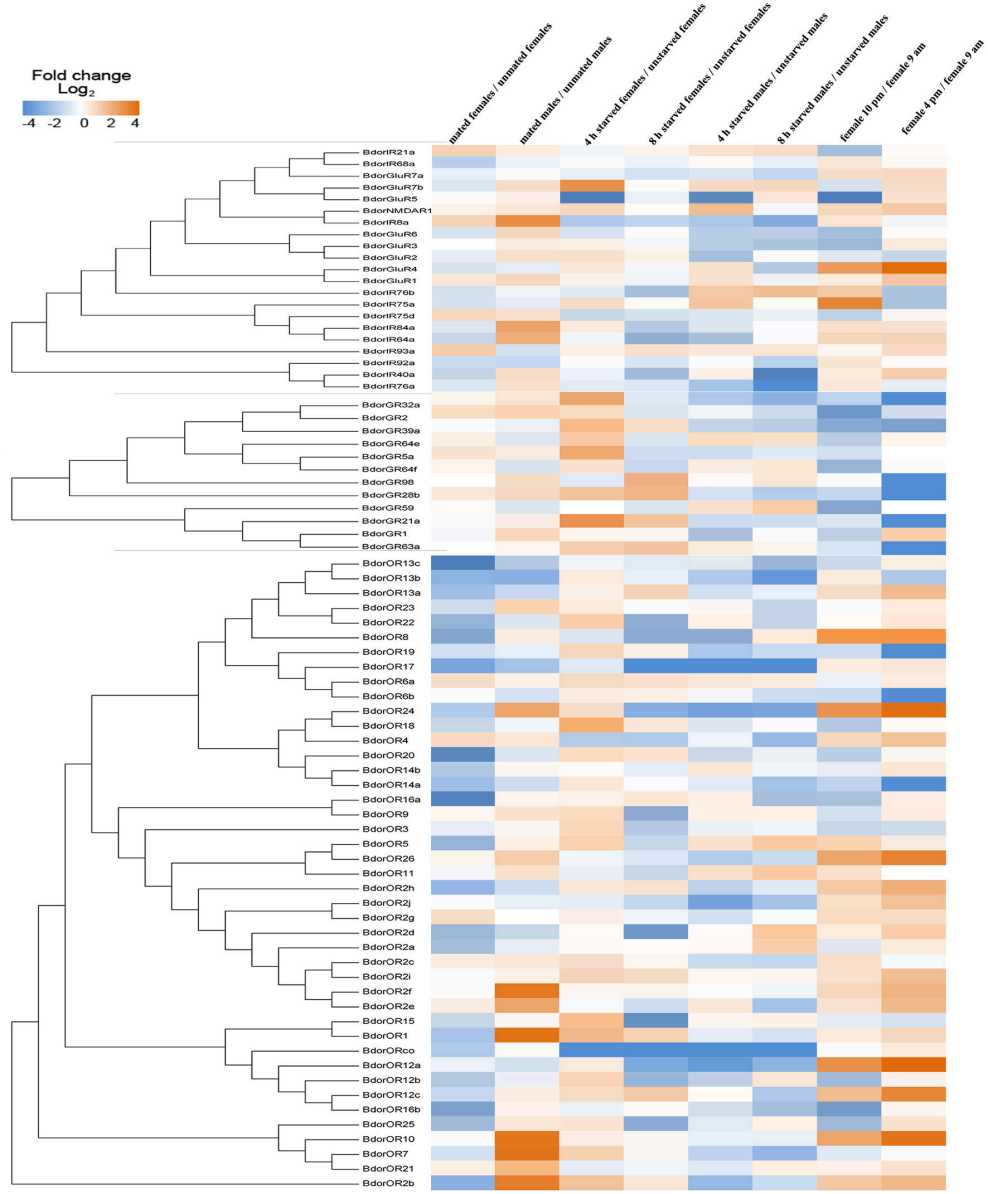
Changes in the expression of chemosensory receptors in different physiological states. The tree was made with the maximum likelihood method with multiple alignments of amino acid sequences of BdorIRs, BdorgGRs, and BdorORs. Bootstrapping supports are indicated beside the branches at 1,000 simulations. The expression of chemosensory receptors was significantly higher (orange) or lower (blue) at different physiological states; non-differentially expressed chemosensory receptors are denoted as zeros (white). All measurements were made with the log_2_ fold change scale.

Mating behavior decreased expression of most ORs in female flies, and increased expression of most ORs in male flies. Expression of GRs and IRs did not show significant change after mating in either female or male flies. Starvation for 4 h increased expressions of many ORs and GRs genes in female flies, although expression decreased when the female flies were starved for 8 h. However, the scenario was different for male flies: starvation for 4 and 8 h generally decreased expression of many chemosensory receptors. The expression of one cluster of ORs and most IRs showed an upward trend over three time points during the day, whereas the expression of all identified GRs showed a downward trend (Figure 7).

## Discussion

In this study, we focused on three crucial families of chemosensory receptors: IRs, GRs, and ORs. Using RNA-sequencing and *de novo* transcriptome analysis, we identified 21 IR, 12 GR, and 43 OR genes in the antennae of *B. dorsalis*. Previously, Wu et al. performed transcriptome sequencing on mixed tissues at different developmental stages (egg, larva, pupa, and adult) of *B. dorsalis* and reported 23 ORs, 6 GRs, and 11 IRs (Wu et al., 2015). Liu et al identified 12 IRs and 35 ORs in the transcriptome of the antennae of male and female oriental fruit flies (Liu et al., 2016). In comparison, our analysis of the antennae not only covered nearly all previously reported receptor genes (Supplementary Table 2), but also identified a number of new chemoreceptor genes. To our knowledge, many chemosensory receptors were annotated and reported for the first time in this species of fly, however, some receptors may be difficult to be identified by transcritome analysis. This may be partly due to the limit of quality of transcriptome sequencing annotation, and the fact that many chemosensory receptors are expressed in the tissues other than antennae (Rinker et al., 2013).

Behavioral responses and sensitivity to plant odors, sex pheromones, or oviposition cues may be influenced by mating status, which indicates that the expression of chemosensory receptors may change before and after copulation. Our results showed that expression of most IRs and ORs decreased after mating in female flies, but not in male flies. This indicates the potential role of these chemosensory receptors in recognition of sexual signals in female flies, as olfactory responses to sexual signals are switched off very rapidly after mating. In a previous paper, OR19 was identified as highly expressed in male antennae (Liu et al., 2016), which is consistent with our results that BdorOR17 (same gene as OR19) is up regulated in the antennae of starved males compared to females. This result suggested its important role in male flies. The mating-related changes of receptor expression were more obvious in female flies, which is consistent with the observation that the female flies have decreasing sexual receptivity and increasing urge to find appropriate place for egg production after copulation (Krupp and Levine, 2014; Hussain et al., 2016). Many mated insects are more attracted to food odors compared to unmated ones (Hussain et al., 2016). The observed increase of expression levels of many receptors in male flies may account for the increased attraction to food signals after mating behavior. In this study, expression of almost all GRs was up-regulated by mating, which indicates the important role of GRs in the process of courtship. In previous studies, GR32a was suggested to function as a pheromone receptor of a male inhibitory pheromone (Miyamoto and Amrein, 2008), and GR39a plays a role in sustaining courtship behavior in males (Watanabe et al., 2011). Our results also showed that the variation pattern of IRs is different in male and female flies, indicating different involvements of these receptors during mating between female and male flies. For example, IR52c and IR52d played a role in male mating behavior and sexually dimorphic expression in neurons of the male foreleg to contact female during courtship (Koh et al., 2014). Further study needs to illustrate functional difference of IRs in male and female during courtship and mating.

Olfactory behaviors vary at different times of the day, which helps the insect respond well over the time (Gadenne et al., 2016). The biological clock is a cell-autonomous system that coordinates physiology and metabolism to align behavioral processes with the day/night cycle (Bass and Takahashi, 2010). The rhythm is important for reproduction through its effect on ovulation (Zhang et al., 2017). We observed that the egg-laying peak occurred at 4 p.m. for the female *B. dorsalis*, consistent with a previous report (Yang et al., 1994). *Drosophila suzukii* has a peak in oviposition activity at 8 p.m., whereas this peak pattern occurs from 4 p.m. to 4 a.m. in *D. melanogaster* (Lin et al., 2014). The peak in expression of chemosensory proteins may correspond to the time of increasing chemosensory activity to odor signals (Rund et al., 2013). Expression of one cluster of ORs and some IRs showed clear up-regulation at 10 p.m. and 4 p.m. compared to 9 a.m., which suggests that these ORs may be required for the recognition of oviposition cues. One oviposition-related chemical in *Anopheles gambiae* was culicine water, which could negatively influence the peak oviposition time (Sumba et al., 2004). The relatively higher expression of some chemosensory receptors at 10 p.m. may be the result of the retention of mature oocytes at the beginning of the night (Allemand and David, 1984). The highest expression of most GRs in gravid female oriental fruit flies occurred at 9 a.m., before peak oviposition, which suggests that GRs may play a role in the recognition of stimulants and subsequent signal transduction inducing oviposition behavior (Ozaki et al., 2011). The roles of some GRs in oviposition process were documented previously. For instance, GR5a can detect trehalose (Chyb et al., 2003), and function as a receptor for caffeine (Moon et al., 2006), GR21a and GR63a can mediate CO_2_ detection (Jones et al., 2007), and GR68a acts in pheromone reception in courtship behavior (Bray and Amrein, 2003). Besides the conventional role of GRs in insect chemoreception, GR28b also acts as a thermoreceptor to mediate warmth-sensing (Barbagallo and Garrity, 2015). Therefore, it is also very important to investigate the changes of GRs expression in response to thermal variations. Our current study indicates that the biological clock plays an important role in the regulation of insect chemosensory receptors expression levels, especially for ORs and GRs.

The nutritional state of the insect can also influence olfactory behaviors. A blood meal could regulate sensitivity to many kinds of odor in blood-feeding insects, including odors from food—resource and oviposition cues (Gadenne et al., 2016). Starvation affected sensitivities of all ORs and IRs in all sensillum types (Farhan et al., 2013), resulting in increased behavioral and physiological sensitivity to food blend (Root et al., 2011). In this study, we found that starvation induced decreased expression of almost all candidate chemosensory receptors in male flies. In contrast, the expression levels of most chemosensory receptors increased after 4 h starvation treatment and decreased with the longer starvation time in female flies. Previous studies showed controversy over the starvation effect on physiological responses. Root et al. suggested that starvation could increase the activity of olfactory sensory neurons (Root et al., 2011), whereas Farhadian did not find any effect of starvation on olfactory sensory neurons that expressed OR47a (Farhadian et al., 2012). In one study, starved flies were more sensitive to odors, a response regulated by short neuropeptide F receptor (sNPF) (Root et al., 2011). Another study showed that starvation increased the sensitivity of olfactory receptors cells to odors, whether they expressed sNPF or not (Farhan et al., 2013). More evidence is needed on the effect of starvation on recognition of chemicals. Although changes in mRNA expression might not necessarily be sufficient to explain behavioral changes, the observation of differential effect of starvation on the expression of chemosensory receptors between male and female flies suggests gender-specific difference of olfactory recognition in response to starvation.

In conclusions, we investigated variations in the expression of chemosensory receptors in different physiological conditions. The results in this study provided us an overview of expression patterns of chemosensory receptors in response to different physiological changes in oriental fruit flies. Our study could lay a foundation for further functional validation of specific chemosensory receptors in different physiological processes of insects.

## Author contributions

SJ, XZ, and FG performed the experiments: XZ analyzed the data, and XY and GZ wrote and revised the manuscript.

### Conflict of interest statement

The authors declare that the research was conducted in the absence of any commercial or financial relationships that could be construed as a potential conflict of interest.
