# Supplementary material for: Olfactory Plasticity: Variation in the Expression of Chemosensory Receptors in Bactrocera dorsalis in Different Physiological States
Source: Front Physiol. 2017 Sep 14;8:672. doi: 10.3389/fphys.2017.00672 (PMC5603674; doi:10.3389/fphys.2017.00672)
Supplement: Supplementary file 1 [file Table1.DOCX]

Supplementary Table 1 Primes used in this study

| Name | 5’-3’ | annealing temperatures | Name | 5’-3’ | annealing temperatures |
| --- | --- | --- | --- | --- | --- |
| BdorOr10-F | AGCAAACTGGAAGGTATGAACG | 58 | BdorOr14b-F | GGTAACCGAAACTGGAGGCA | 59 |
| BdorOr10-R | AAATGCAAACATCGGCAAAG | 58 | BdorOr14b-R | CAAAATGCTCAATGCGATGTG | 59 |
| BdorOr7-F | TTCGGAAAGGCAGCACTACAG | 59 | BdorOr14a-F | CCATAGCCATCAACGACAGG | 59 |
| BdorOr7-R | AAGAGCAGCGAGCAAATAACG | 59 | BdorOr14a-R | GTTTACCACGATTTAGACCCAACA | 59 |
| BdorOr17-F | GCCACCGCCAATGCTATGTT | 60 | BdorOr19-F | TCGTTCTACGACCTATGGTCTAGC | 56 |
| BdorOr17-R | CTCGCACTCAGTCGTCAATCTTCT | 60 | BdorOr19-R | AATTAAGCCCGCACAAAAGC | 56 |
| BdorOr5-F | TTCTACAGGAGGGCATTGATTC | 58 | BdorOr9-F | TATTGGACCAATGCTCCAGTCA | 58 |
| BdorOr5-R | CGGATAGGTGTCTACCGAGCA | 58 | BdorOr9-R | CAAAATTGTCCCGCAAAAGTG | 58 |
| BdorOr2j-F | ACAGGAAATAAAAGCCAGGAGC | 59 | BdorOr20-F | TGCGTTCTACAATCATCCGTG | 59 |
| BdorOr2j-R | TTGTAGCGAAGTCAGCGAAAA | 59 | BdorOr20-R | AAGCCGTCAAGTGAGCGTAA | 59 |
| BdorOr2h-F | CACAGATGCTACGAAGAGTAAGGA | 60 | BdorOr8-F | AGCAACCATCAGCAACACCA | 57 |
| BdorOr2h-R | AGAGTCGAAGCGATTTGGGT | 60 | BdorOr8-R | TTCACCACATCGTAACATCTCCT | 57 |
| BdorOr2g-F | TCCAACGCAGGAGGATAAGG | 59 | BdorOr25-F | TGATGTCCATTCTACAGGGCTTAT | 59 |
| BdorOr2g-R | GGCGGAGACCACAGTCAAAGT | 59 | BdorOr25-R | CGTTCATACCTTCCAGTTTGCT | 59 |
| BdorOr2c-F | GCATCGTTTCGCATCAAGAA | 55 | BdorOr21-F | ATCTATGTAGCCGCTGGAGGA | 59 |
| BdorOr2c-R | CATGTTAAGCATACAGACGCAGAG | 55 | BdorOr21-R | GAAGAAGGGTGTTTGGAGGG | 59 |
| BdorOr2i-F | CGATCCCTTCCCTCACAACG | 61 | BdorIR21a-F | AGGTTACAACGACGCATAGACAG | 60 |
| BdorOr2i-R | TGATAACAGGTCGGCACAGTCT | 61 | BdorIR21a-R | AAGAGGCTTCCGAGGACAGA | 60 |
| BdorOr2b-F | CGCTCTTTGGTATGATGCTATG | 58 | BdorIR40a-F | ATGGCAAGGGTGAGGAGAAC | 59 |
| BdorOr2b-R | TTGATTGAGACCTTGTAGTAGGGA | 58 | BdorIR40a-R | CCACAGATTAGGACGATGAAGG | 59 |
| BdorOr2f-F | TTCACAATAGCCGCSACCRTACT | 60 | BdorIR92a-F | GTTTTCATACAACAGCCATCCG | 58 |
| BdorOr2f-R | CGCAGTCCAACTCRAGCATC | 60 | BdorIR92a-R | GCACAGAGTATCATCGCCAGA | 58 |
| BdorOr2d-F | ACGCTACAAAACGCCACAGG | 59 | BdorIR76a-F | GCCTTCTCGACGACGATGTT | 59 |
| BdorOr2d-R | GAAAAGGATACCAGCCACCAAA | 59 | BdorIR76a-R | GGCACGGTGAGTATGCTTGA | 59 |
| BdorOr2e-F | GAGCTGATCTTTTCACGTTTGG | 58 | BdorIR68a-F | GTTTTGGGAGCAAGGGAAGC | 59 |
| BdorOr2e-R | AATATGACGCTGCTGAGTAGGC | 58 | BdorIR68a-R | CGAAGACAACAATGGGCATG | 59 |
| BdorOr2a-F | TTAGGCACGAACTCCGTCTTT | 57 | BdorIR75d-F | TCGCCACATTTGCACGAGAT | 57 |
| BdorOr2a-R | GCAGTATCTGCTGATGCGATG | 57 | BdorIR75d-R | GCGGAGCGGTTGAGTAGGTT | 57 |
| BdorOr26-F | ACTAATCCAACACTAACGCAGGTA | 59 | BdorIR84a-F | GATGACGCCGCTGCTACTGA | 58 |
| BdorOr26-R | CACGGCCAGTATGAAGAAGC | 59 | BdorIR84a-R | CGCCTATTGACGAAGGTTTGAT | 58 |
| BdorOr12a-F | ATAGATCGGCAGTTGAGGCA | 57 | BdorIR64a-F | ACAAGTTTCTTTTCGGGTCGT | 60 |
| BdorOr12a-R | TTCACGAAGAAGGCGGTAAA | 57 | BdorIR64a-R | TATCGTTTTCGGCTTCTCCA | 60 |
| BdorOr12b-F | TTAGGAACCTGCATCTGCATTA | 56 | BdorIR75a-F | GCTTTGCTGCTCCTACTAACGG | 59 |
| BdorOr12b-R | AACATCGGGTAGCCAGTGAAA | 56 | BdorIR75a-R | AATACCATACGCCCACCCATA | 59 |
| BdorOr12c-F | ATAAAGAAGACGCCGAACTATGTG | 59 | BdorIR76b-F | GAGCGTCAGCTACGAAACACC | 62 |
| BdorOr12c-R | GCAATGAAGACTGACGGGACA | 59 | BdorIR76b-R | GCCCACTTGCTACCCTCATTAC | 62 |
| BdorOrco-F | GCCATCGACTCTGCAAACCTT | 59 | BdorIR93a-F | CACGAACAATGTGACGGACG | 58 |
| BdorOrco-R | GCCGCTCGACCCAGTATTTT | 59 | BdorIR93a-R | GCAGCAAACAGATAAATGCGACT | 58 |
| BdorOr4-F | GTGCTCCAGTCCAGCCTCAT | 60 | BdorNMDAR1-F | GGTGAAAGGCTCGTCGGTAG | 61 |
| BdorOr4-R | AAGCCAAATAGCGAATCCTCA | 60 | BdorNMDAR1-R | GCGAAGAGTCCCAAATAAAGG | 61 |
| BdorOr6a-F | TCGGTCAAATGCTGATGGAT | 61 | BdorIR25a-F | CAGGATTGGACTCGCCGTTAG | 58 |
| BdorOr6a-R | CGTGCTGTAAGTGAGTGGAAAA | 61 | BdorIR25a-R | CTTTGGAGCCTTCTCATCTTTG | 58 |
| BdorOr6b-F | CCTCTACTACCAAATGGGCTCC | 58 | BdorIR8a-F | AGGGCACTAAGGATGAAGGACA | 61 |
| BdorOr6b-R | CGGGTTTTCGTAATATGTGCTG | 58 | BdorIR8a-R | GTTCGATTAAGCCGCGTAAGA | 61 |
| BdorOr15-F | ATTACAAGAAGCGGGTGCCA | 57 | BdorIR100-F | GACGAATGATACGCTTTGAGACT | 58 |
| BdorOr15-R | CCCAAATCATGCTAACCAACG | 57 | BdorIR100-R | ATAAAGGCAGTACGGATTGAGG | 58 |
| BdorOr1-F | ACACGGTGGATTTACCATTTCT | 56 | BdorGr1-F | TGGGGTGTAGCAACAGAAGTG | 59 |
| BdorOr1-R | AACATTCACGCTTTCATTCTGC | 56 | BdorGr1-R | ATGCCAACGTATGCCACAAT | 59 |
| BdorOr3-F | GCGGATGCGTGACTTGGTGT | 61 | BdorGr21a-F | TCCGCCTCACTGGAACTAACA | 59 |
| BdorOr3-R | GCAACGGCTTGAGATTGTATGAG | 61 | BdorGr21a-R | GTGACTTAAATCCACCCAAAGATG | 59 |
| BdorOr24-F | AACACCATACTTCGGGCGTAC | 59 | BdorGr28b-F | GGCATTGAGGCACAGGAAGT | 59 |
| BdorOr24-R | TGCTTCCAAAGGGCAAAACT | 59 | BdorGr28b-R | ATGAAGAACGGCGTGAGACC | 59 |
| BdorOr18-F | GAACACCATTGCCTGCTCCA | 59 | BdorGr98-F | TTCTGGCTTTGCTCATACCCT | 57 |
| BdorOr18-R | TTTGCTCTGCTCCGACACCT | 59 | BdorGr98-R | CGTGATTGTTAGACTTATCGCTCC | 57 |
| BdorOr23-F | CGACACGCAAGTGGGTTATC | 59 | BdorGr39a-F | TGCCACCGCTGCTGATCCTA | 61 |
| BdorOr23-R | CTATTTTCAGCAGCAGCCTCA | 59 | BdorGr39a-R | CCATTTTGTCCAATCCAGTTCCTG | 61 |
| BdorOr22-F | TTCATCTTCACCGCAACCAC | 57 | BdorGr32a-F | GCAAAATGGCATGGCAACTA | 60 |
| BdorOr22-R | GCAACACGCATTCATTACCTTA | 57 | BdorGr32a-R | AACATCCCTATCTCGTCCTGGTA | 60 |
| BdorOr13c-F | TGCGTTCTACAATCATCCGTG | 59 | BdorGr2-F | GCACCCAAAGGGCTACCAAT | 59 |
| BdorOr13c-R | AAGCCGTCAAGTGAGCGTAA | 59 | BdorGr2-R | AATGAAGGGCGACGACAAGG | 59 |
| BdorOr13b-F | ATCAGACCCACCGCATCCTT | 60 | BdorGr5a-F | ATACGGGCCTCACCATAAACA | 58 |
| BdorOr13b-R | GCAATACGCTGCCCAACATAC | 60 | BdorGr5a-R | GCCAGTCGCAGGAAACCAAT | 58 |
| BdorOr13a-F | TGGACCATTACCTCGCACAT | 58 | BdorGr64f-F | CAACGGCAAATAGATAAACCACC | 58 |
| BdorOr13a-R | ACCAGGCCAGTATTTCAGCAT | 58 | BdorGr64f-R | ACCGACAGCCTGATGAAAGC | 58 |
| BdorOr16a-F | CCATAGCCATCAACGACAGG | 59 | BdorGr64e-F | CAACGGCAAATAGATAAACCACC | 58 |
| BdorOr16a-R | GTTTACCACGATTTAGACCCAACA | 59 | BdorGr64e-R | ACCGACAGCCTGATGAAAGC | 58 |
| BdorOr16b-F | CCATAGCCATCAACGACAGG | 59 | BdorGr63a-F | AAAGCTCATTACGCCTCCTTC | 57 |
| BdorOr16b-R | GTTTACCACGATTTAGACCCAACA | 59 | BdorGr63a-R | GTCCGAGTTCATCCAATTCAGT | 57 |
| BdorOR59a-F | CAACTGGGTGGAGCAAACAC | 59 | BdorGr59e/f-F | ACACGAAAACCTGTAAAGAACTCC | 59 |
| BdorOR59a-R | TGATGACAGCCAACAGGGAG | 59 | BdorGr59e/f-R | CCAATCTCATAGGCACCAAGC | 59 |
| BdorOR69a-F | GATCTGTTTGATGGGCTTCTCC | 60 | BdorGr32-F | GCAAAATGGCATGGCAACTA | 60 |
| BdorOR69a-R | GCTCGTTTCCGTTAGCTCCTT | 60 | BdorGr32-R | AACATCCCTATCTCGTCCTGGTA | 60 |
| Bdor-tub-F | CGCATTCATGGTTGATAACG | 58 | Bdor-tub-R | GGGCACCAAGTTAGTCTGGA | 59 |
