# Supplementary material for: Olfactory Plasticity: Variation in the Expression of Chemosensory Receptors in Bactrocera dorsalis in Different Physiological States
Source: Front Physiol. 2017 Sep 14;8:672. doi: 10.3389/fphys.2017.00672 (PMC5603674; doi:10.3389/fphys.2017.00672)
Supplement: Supplementary file 3 [file DataSheet1.DOCX]

>BdorGr1 Cluster-473.0_m.439

MNNQRMDTPNLLLSEIKLKKWKNYDNKYSNSSLNGTLKDYSFHSTENETIERYDQVTIYSLVISAKQTFYRDHKLLLVLFRVLAVMPIQRSSPGRMTFSWNSTATLYAFIFWIFMTIIVLIVGRERIQILYTTKQFDEYIYAVIFVIYLIPHFWIPFVGWGVATEVAEYKSSWGKFQLKFYRVTGTTLQFPHLKSTIIIISIGCILCAFLFLFALSFFLEGYPLWHTLAYYHIIIMINMNCALWYINSRAIKTASIALAHSFQKEILISYSADIFSKYRMLWINLSELLQSLGNAYARTYSTYCIFIFVNIVIAVYGAFAEIFDHTNLSRDSYKEFGLIVDGLYCSILLFIFCDCSHNATLCVAKGIQNVLLKIDVRQINRKAKSEIDLFIFAIEMNPAIVSLKGYVNVNRELLTSFIATITVYLLVLMQFKFTLN

>BdorGr21a Cluster-1561.0_m.1377|Cluster-48863.0_m.44749

YREKLEILAENNTISTDLFVRKFEDIDDPVLLDKHDSFYHTTKSLLVLFQIMGVMPIHRNPQKPGMPRTGYSWTSKQVFWAVCVFSMQTTIVVMVLRERVNTFLNDSDRRFDEAIYNVIFISLLFTNFLLPVASWRHGPQVAIFKNMWTNYQLKFLKVTGSPIVFPNLYPLTWSLCFFSWGVSIAINLSQYYLQPDFKLWYTFAYYPIIAMLNGFCSLWYINCTAFGTASRALSASLELTLMSDKPAKKLTEYRHLWVDLSHMMQQLGRAYSNMYGMYCLVVFFTTIIATYGSLSEIMDHGATYKEVGLF

>BdorGr28b Cluster-17584.0_m.16010

KKLKESIFGYINTFLHITIYVACYMLTLINDFETVAGYFFNSGVSRFGDTLQIFSGLIGVTIIYITAMLPKQRLEYSLRTVQDIDLMLHKVGVKIIYTKLLHYSYFSILLVVAVDTVYSCGNFMLLKSANLEPSTPLYVVFTLQHTVISIATMMYHGFVKMLEMRLTMLNEVLKKLAHQWDNSIVKPMPKQRSLQCLDSFSMYTIVTNNPCEIIQESMEIHHMICDAASTANKYFTYQLLTIISIAFLLIVFDAYYVLEILLGKSAHEGKFKTVEFVTFFSCQMILYVIAIVSIVEGSNRAIQKSEKTSGIVHSLLNKAKNAELKEKLQQFSLQLLHLKIHFTAAGLFNIDRTLYFTISGALTTYLIILLQFSNSNVPEHPFPPMEENDTTPIRSLVSNLTIGG

>BdorGr98 Cluster-45566.0_m.41606

MGLMEFSESTLIAVIAPYLWTFSLFAVVMPPYYILRAVPTNRHWMLYIIRTVFVLYMLAQFVISFWVAYTYNVLLSGFLMRNSLDIMTCVLSIGINIVQLAVQMTVYVQALTKHNLLRDVLNDIVQLESDIRKHLTTDCSLASFRWRLGLRVGIWFVVVSTFVPYLSYMLYTQYFHPIKRGIIVFFGTIIHFKGVEYCISVQTIQELLQLVQQQLVHLRRELVRCERVELRFSLYDDLQTNQKLLARVWNLLNQIERYFFIPMLMLFFINGFAIIQAIHWAYINFERDNLNLRLYRIIYTVMIILALLIPCYLSQCCIDEYNRFGTILHKLKTMGIDEQLNMRLQEYSLQLMHQKMLFTCGGFFDINLKNFGAISLTITTYIVILIQFKLQAETEKKSNIGIRFE

>BdorGr39a Cluster-30840.0_m.27945|Cluster-32529.0_m.29524

FRMATLKYWQIYLPLMRLVGICPASFSPENKKFVSTARDLIIALSITTLTMIMMECCLLISIHLAFSGDLNMFDDFKTANTLFLVQIITATVLQLFQSIYIFVSRNTGIEMLNVLYKHVCVYSEQFLAGDFHIDRCVRWTLIFHIVIFVVFEFNLLYSIWMPNLPLWLILCLSFVLTIRPTYCFIMISTYIYIVHVLCHILAVYNKRLQAIEIHLIKSKIAVRLKLYELLILRAELISLVCKHMRQVYGVPMLLETCYVLSVLPSFPAFVLKMISWNISFNIVFLKYCCTALIWVLPPLLILIQAMAANTINAEANKTVKILAKIPHTGTGLDKMVDKFLMKNIRKKPILTAYGFFQLDRSALFKLFTAIITYIMILVQFTDIENSLKTKEIQTNIN

>BdorGr32a Cluster-27473.0_m.24936

MSSNKVKPSPIQRHHNGLNQFLRQPPTHNSIFKDMRITLCVLKATGLLPIYEEVSSYEVGPPTKPKIYYSFFIRGVVQTFTLFNLYNLVTPGSTGQLFYSYSDTDNVNKWIEYLLCMLSHSATVIICGRNSKLFIKILNEILKVDEDVFDRFRETLKNKCGFSLKYIVGICICQWYLIVLRVLAVKDTLNVNSYIFLFIYAVQNGMATIFIVFTAALLRILKMRFAHINTTLKGYTYSEQHKLRRIPGRDRDVITMDSFPEESLFIYRLHNKLLRIYRSINDCCSLILVAYMGYAFYTITTTTYNLFVQITTQRLSFNVLQTCFVWLAMHTCVLALLSKNCGQATDEANGTSQVLARVYGASKDHQNIVDKFLTKSIKQEVQFTAYGFFIIDNSTLFKIFSAVTTYLVILIQFKQLEDSKMEDDWSKL

>BdorGr2 Cluster-55772.0_m.51037

FKRRTSKLLIVFVKMRSLNRVGHESNAKSVKTRKPAKNSILLYLKCHLLLLKYLGLLPFYTTLSAYEIGMPTQRSLYINRVILLAKFALNISHINAVLSPIILQLLFTRSKTDGITNLLDVVFCMLSDITITWTCARSTTEILLIINSFLRVDKLLKQHPDSPAERSCATNHFNRYLFLVFGYISLVMIAYVKHTLDYFSIYFCAYITFYQLENAISCGFVVFISALLHLLTERFQYVNQLIEQYTSKNLVQKRYPYTSTVSILSFNRNTNMHDDAIMRMFAQNSATIYSLHIDLLDIYKMINKFAGLGLLMFLLYACYGLLSCAYGCYVCEWQRSDDLYYGIWTFSWIPLYAGIIILLATNCAKATNQANNTSKILARVYGKGKEYQNIIDKFLTKSIRQDVHFTAYGFFVIDNTTLFKISSALVTYLVILIQFKQLEKSKD

>BdorGr5a Cluster-17902.0_m.16305

IAQCFCLMPVRGILAASPKGLSFRWKSFRTWYCILYTLVTIADTGLTINMVVKGVLDVRNIEPLIFHANILLASIGFLRLAAKWPQLMRKWQRVERHMPPFQSWREREALAVRVHKVTFVLITLSLTEHLLSTISAIHFANYCPSRVDPIESYFMTVVSQIFFVFDYSTWLAWFGKILNVLMTFGWSYMDVFLMIIAIGLSSLFEQVQHSLERVKGQVMSESFWTRTRLQYRLICDLIEQVDSAVSAITVLSFANNLYFVCIQLLKSMNTMPSVAHFVYFYASLCFLLARTLAVSLYLSEVNDRSREPLKIIKKVPKEGFHPEVDRLAHEIGMDTVALTGLKFFNITRGLVLTVAGTIVTYELVLIQFHEDQNLWNCN

>BdorGr64f Cluster-4771.0_m.4404

MKFAFKIGWKIGNVAADARLRVQQQHDARKNRRLWRRQCERGIRRLSQNGSNAAATCGPQKPNPKSTLIRRTCTEFVAQRELMQLQIDKPPKILAKSVKEDFQHDGSFHQAVGKVLLFAEFFAIMPVKGVTAAHPRQLSFSWTNIRTLYCLLFITTTTIDLGLTMNKVLHKPINFDSVEPLIFRLSIIVVCISAIVLARKWPALMLDWYEIECDLPEYLTQMEKGRLAYRIKMVTVVAMTLSLGEHLLNILSNIHYSKYCPQTEDPIENFFILTNQHLFMIFSYSVPLAIWGKVQNILCTFIWNYMDVFVMIVSIGLAAKFRQLNDNLFKFKGMRMPEMYWSTRRKQYRNLCELCTRIDGAISLITMISFSNNLYFICVQLLRSLNKMPSLAHVAYFYFSFFFLIGRTLAVSLYSASINDESRKPLRVLRCVPKESWCTEVKRFSEDISTDLVALSGMKFFYLTRKLVLSVAGTIVTYELVLIQFHQDSKLAECVSSIRTLSSPGNNTLH

>BdorGr64e Cluster-17630.0_m.16054

MKVAANVTKNWLQRQQVRLQNIKVWQRGRKVGSDLLEIKEWSAKPKSAKQRKRQIGMVRVRNLFRRGTKKDYEHSGSFLEAIGPVLLLAQFFALMPVCGILSKTASKVYFSWKSVRTCYAMLVIFCLGPASLCTIAFAFRERFNFDTVEAIVFYVSIFLIAMAFFQLARKWPALMVKWESIESKLPPLKTEMQRAALAHRIKMITLVATMCSMVEHLLSMLGIIYYVNACPTMPGHPIRSFLYTNWSQYFYFFDYTDWAGIFGKVLNVISTFAWNFNDIFVMAVSVALSARFRQLNEHMLRVAKRPTSEKFWIENRINYRNLCKLCEATDDTISLITLLCFSNNLFFICGKILKSLQKKPSFSHTMYFWFSLGFLLMRTLMLSLYSAEINDESKRPLVVFRSVPSVSWCRELKRFSEEVTTDVVALSGMKFFHLTRGLVLTVAGSIVTYELVLLQFNKEGKVNDCYEG

>BdorGr63a Cluster-34143.0_m.30992

MFNSYNRRKKHDTVFLNVKPTFNGQGNGLRKYSTGLLDKEDNPFYDVNSSSGSRASVGTITTLNENFRANIFYNNIAPIQWFLHMLGVLPITRREPGKAKFRINSIAFGYSFAFFILLSVFVTYVAKNRISIVTSLSGPFEEAVIAYLFLVNILPLILIPILWWEARKIAKLWNDWDDFEILYYQISGHSMPLNLRRKTTMIAVVLPILSILSVVITHITMADFQIIQVIPYCILDNLSAMLGAWWFIICESLSMTANILGERFQRALRHIGPAAMVADYRALWLRLSKLTRDTGNATCYTFTFINLYLFFIITLSVYGLMSQLSEGFGIKDIGLAITAIWNVFLLFYICDKAHYASFNVRTNFQKKLLMVELNWMNSDAQTEINMFIRATEMNPSNINCGGFFDVNRNLFKGLLTTMVTYLVVLLQFQISIPTDTGRHMNVSVAELATDMMLESAEDELTTTSTTSTTTTTTTKMPPPARGRKG

>BdorGr59e/f Cluster-39213.0_m.35652

MKIIKDMKYVNFVGQVFGLVPIYEVKRGELRISLRGHYFTYFINILLFMISLTVSWFVLNSDSGFSVFRNLEGSDQTTEALFCLISCNIIIFVSSTNSRRYCGILQEIGKMDAYLVAKGFTAAYTCYLLTCFLIITAAGLLYTAYYYMDMLSDHFHYHQAILLGIYSLQLLISNLYAICLRVLLGNISKRIDFVNGQLEISTKSDLAVESNWR

>BdorGluR3 Cluster-11313.0_m.10311

LRYDMEMNNNYKSVLRRVKKLGDNHIVVTGSSDTMPEFLKQAQQVGIMNEDYKYIIANLDFHAFDLEEYKYTEANITSFRLFSPEQKAIQEIMEKMGHKSSLEDLRNGSCPITVSMALTYDSVQLFAETTKHLTVRNVPLNCSDRSESVLDDGSSFKNYMRTLKLQKRTLTGPIYFDGNVRKGFTLDIIELQPSGIIKIGTWDDISNLTIQRVAQTNSVIDNVDNSLANKTFVVLLNVPNKPYASLVESYEKLEGNSQYEGYGVDLIKELAQKLGFNFILKDGGNDYGSYNTTTNTTSGMLKEIIKGRADLAITDLTITAARQQVVDFSIPFMNLGIAIIHVEPQKDTQAFFTFMDPFSQGVWWLLGLSFLFVSFSFFILGRLSPSEWDNPHPCIEEPSELVNQFTIGNSLWFTTGALLQQGSEMEPKAISTRTVASIWWFFTLLMVSSYTANLAAFLTIENPTSIINSIDDLVAEKVAYGAKKIGSTREFFEKSEDPRYIKMNDFMNANPKYLTDSNTEGVNRVDKGYAFLMESTSIEYHTMRMCNLSKIGNALDEKGYGIAMRKNWPYRDKFNNALLQLQEQGTLEKMKNKWWNEVGAGICSSGKQRSEDTELKMENLSGIYIVLGVGSALAFVYGIVIWFYYIHTKARHYHLSMREVFVEELKFALDFNSYTRVLKNTASMYSRGRNSILSVSSTQDHKSLKD

>BdorGluR2 Cluster-52009.0_m.47628

MQVQNNWIFGILYTFIIYVKGFANIEENEGISIGIISDDNMEPLQKTFNYAITVANTDLGIPLKGYNEQIQFGNSIEGHAKLCKFMQTGIGAIFGPSSRQTSAHLLTVCDAKDVPYIYPHMSENVEGFNLYPNPIDLARILHDIINLFEWTHFTFLYESSEYLSILNGLMPFYGSDGPIINVLRYDLKLSGNFKAVLRRVRKSEEGHIVVVGSTPSVAELLKQAQQVGIMNDKYSYIIGNLDLQTFDLEEYKYSEANITGFRMFSPTQAIVQELISQLEMDYNENNNNQIANGSCPITLEMALTYDAVQVFAESTKNLVYRPQALNCSEQSNQVQADGSTFKNYMRSINMQEKTITGPIYFDGNIRKGYSLDIVELQTSGLVKIGTWDERNNLTIQRPPQSELWSEVDANSLVNKTFRVLISVPNKPYASLVESHKKLVGNNQYEGYSIDLIKELAAKLGFNYTFIDGGSDYGSFNKTTNKTTGMMKEINEGRADLAITDLTITSEREEIIDFSIPFMNLGIAILFTQPQKSPANNFSFMDPFSRQVWIYLGLVYIGVSFCFFILGRLSPTEWDNPYPCIEEPEELENQFTLNNSFWFTTGAFLQQGSEIAPKSLSTRTLASIWWFFTLIILSSYTANLAAFLTIEKPVGLINNVNELASDTRVKYGAKKTGSTRSFFSTSEHETYKKMNDFMVENPDLLFETNLEGVNRVKTDNNYAFLMESTSIEYHIVRECNLKKVGEPLDEKGYGIAMVKNWPYRDKFNNALLELQEQGVLARLKNKWWNEVGAGVCKKKSDSSEVNPLDLKSLGGVYLVLGVGSGLSLIYSLIMWCIYVARKSNYYEVPFGDAFLEELRIAIDVANKERILKSAQSVYSRSRNSLVSIDSIDTDSEIENSSKIDRESEKTI

>BdorGluR6 Cluster-7745.0_m.7054

MARPCIQSLPQFTTTTRCPRITLILLLALSCLSCLQIAASQKTNVGLIYESDNPDMEKIFQIAIDKANEESGGALELHGIAVAIEPGNAFETSKKLCKMLRQNLVAVFGPTTDLAAKHAMSICDAKELPFIDTRWDFAVQMPTVNLYPHASQLAVALKDLVVALEWTDTFTIIYETGEFLPTVNQLLEMYGTMGPTITVRRYELDLNGDYRNVLRRIKNSGDYSFVVVGSMATLPEFFKQAQQVGLMTDDYRYIVGNLDFQTMDLEPFQHGDTNITGIRLVSPDEKLVQDLAKTLYETEEPFQNVSCPLTTSMALVYDGVQLLAETFKHVMFRAVPLNCNDASSWDKGYTLVNYMKSLSLTGLTGEVKFDYEGLRTDFVLDVIELTMSGMQKIGEWKTEGGFFANRPPPKIVEVDQRSLVNKSFVVITAISEPYGMLKETPAKLEGNDQFEGFGIELIEELGKKLGFTYTFRLQVDNKYGSFNPKTGKYDGMMLEIIEGRADMGITDLTMTSIREEGVDFTIPFMNLGIAILFRKPMKEPPKLFSFMSPFSGTVWMWLGIAYMSVSLTLFILGRISPTEWDNPYPCIEEPTELENQFSFPNCLWFSIGALLQQGSELAPKAYSTRTVASIWWFFTLILVSSYTANLAAFLTIESLSSPIENAEDLANNKGGVKYGAKVGGSTFTFFQDAKYPTYQKMYEFMRDHPEYMTSTNAEGVDRVENENYAFLMESTTIEYITERRCSLTQVGSLLDEKGYGIAMRKNWPYRDMLSQAVLELQEQGVLTKMKTKWWKEKRGGGACSDTSSEGGAVALELSNLGGVYLVLIVGSCFGVLVAFLEMVLGVKERSDENKVSFKTELIEEFRFVMQCSGNTRPVKYPKNSSRSRSRSRSSRSRSHSRSSSKSSTLSVDSLPMDESKLHHISEHTKHAK

>BdorIR21a Cluster-25382.0_m.23090

RLLNMIASKLNFTIDIIEPARRTNVKSVIDNIMLQVRTKAADIGMCGLYITDDRITETDMSIGHSRDCASFITLASKALPKYRAIMGPFQWPVWVCIVVIYLGAIFPIVYSDRLTLRHLIGNWGEMENMFWYVFGMFTNSLTFSGKYSWTSTQKTSTRLLIGSYWLFTIIITACYTGSIIAFVTLPAFPNTVDSVNDLLGLFFRVGTLDNGGWETWFQNSTHAPTVKLYKKMEFVSNLEEGIGNVTQSFFWNYAFLGSAAQLEFMVQKNFSDDNISRRSALHLSEECFALFQVGFLFPRDSVYKRKIDSMILLAQQSGLMNKILNEVKWSMQRSASGKLLQASSANALRERIQEERQLTTADTEGMFLLMGIGYLLGAIALVSEIVGGITNKCRQIVRRSRKSISSAWSSKRNSEDGEGLRTAAEQLAHEQRKEAKRKAEKQGFGMREFNLTKKTLKELYGNYYKQEPTYVLKDGKLLLETEALSTSSADYHSRDSSGEVPANMLPHLHCKKKAMLVAEIDVERERERELMAAAAEESLAALDACLKLEQDTDSSERSDDDVAYEYELFGSLVEPEGPLSTKLDDLNLFTEGAAELEKVENEPEEARES

>BdorIR40a Cluster-21395.0_m.19531

VFHMAYIKEITGDNEMTRRLLRQQQQQQRQGEVQQMRSGRVQGLAEIPNNLFDKCIWFTVQLFLKQSCKELYHGYRAKFLMIVYWIAATYVLADVYSAQLTSQFARPPHEAPINTLQRLQKAMLRDGYQLFVEKESSSLEMLENGTEVFRQLYALMKLQNPDMEGYLIDSVEAGILLIADGLENKAVLGGRETLYFNIQQFGSKTFQLSHKLYTRYSAVAVQIGCPFLDSLNDVIIHLFEGGILDKMTNAEYATQSRMLGKEYNALHPTNPSETNGNNEPPPSDDNRNANGGSDINGKGEESTEATTKSLDSQIIQPLNLRMLQGAFIVLICGYAAATGILVLELCCHRLNSNFMERTQARLLRRYRWCSRKVRRMTHMLFVRIMR

>BdorIR92a Cluster-37102.0_m.33774

ADSALQREYLEAVQLAFRNLSQQGRLIGLQWIDVSQLDVGGGGRGSCRSSSINCGDSYNNTCADNLAYNDELELCVLRAVDIVTEGFITILSDTVRFLHARYFATRNAELRLKDKFYLFFCEHERPEELLSTEILQFYPHHLMVTPETLTAQQSNNQQATTKLTKPTTTTMPLSTIATASPSAHRDINIQLWTQKFVGASGNLEALLLDAFLPNETFARNAELYPNKVNNLRGRTIRVGSITYIPYVVANYVPAGIGDVDALNSSDYSRTISYLGSEAELMKSFCEVRNCHIRLEPYGADNWGYIYENESATGMLGDVYTQNVEVAIGCIYNWYNNITETSNIIARSSVAILGPSPAQFPAWRANIMPFSNALWIFLILTILLCAAVMYLIRFVASLLDKWLRGVQCEFQHLTAFGQATLDMFAVFIQQPSGPTSLNTFAARFFLAMILCATITLENTYSGQLKSILTVPLFTEAVDTMEKWSKTDWTWSAPSIVWVQTIDSSNIEKEQIMAEKFEVRDYDFLYNASFRSDYGLGIERLMSGSFSFGDYVTAPALETKIVSKDDLYFDWTRAVSIRGWPLMPLFDKHIRACVETGLFVHWERKIVAKYLNRQTQEIMLNLASGHINKLPPQKLTIENISGATFTLLFGCLIASFVFVLELTAHYFNKFQGLCIQRNEKSEN

>BdorIR76a Cluster-39368.0_m.35809

MSLLSPAVNHWTALLNIIIQTYFIDSHATCILWHHDFPFELQTPANGEFIQYINIWPDNLSQSLQQDIYNFTAFAETQLAHGMQPDALVQKLTIAIRESHCETFVAFQEDILSFARSFYNASRISVWRSLRNKFLFAYRKDLQQDTTAYFDDSLFIDQPNVLIVEAECGNCSTFALKTNKFIGPLAEHPEQLYVLDRYNGVDGKFELGVDLYMDKVQNLQGREVTVGVFDYRPFTVIDYERQPQIKDHSPENPRGMAHIDGTEVRMLLALCEVVNCTVNTDTSEDDWGTSYANLTADGIFGLVTSRKSHYAVGALYFWPDDYRYLDMSLFIGRSGVTCLVPSPHRLTSWLLPLRPFQPTLWLGVFACLGVEALALFFTRHLAPSDTEPQYGLMESFQFGYITTLKLFVSQGSDYVVNSHTVRMVLFACYMMDTIVTSVYGGGLSAILTLPTLEEASDSVERLYRHGIPWTATSPDWVISLKGADDDPMVEKLLQKYHVYTYEQLTEFAKTENMGFILERLAFGHFGNVDFLTDESFKRLKLMIDDIYFQYCFAFVPRLWALLPKLNDVIMRVHSTGLDIFWEWEVAATYMDGQQQEEIQASMYMDFDVGPVKLDMGNFIGLVLPLIIGFVFSIFTFIGELIYYKYTQKKAQAVINVN

>BdorIR68a Cluster-37927.0_m.34491

MVRVLKQIRKQNCEFMLVTLLNGLQVQRFLRFVEKNRLLNLQQRFVLLEDSRLMAAEMWYIWSNMISTVFVKPLDNQRFLLNTIAYPEILNGVVVTKRLMFWEQGKHIKINQLFQDRTSNMKGFAMPIVVFEHVPMVRRHTDNATLVGLEVEILKSLGVKLNFKPDFYETNDAAYEHWGKELPNGSYSGVLGDMANRNARIAIGNLHMFKVYASVMDFSWPHSFECLTFLTPESSQDDSWRTLIQPFSGSMWAGVLFSLFVVGTVFYVISCLHAILLHRRSRRTPRILNWSEWRSKGLLRPSGSVDTKLFRDVQFRRYLGQLKSLPLKNTDLFDDYSNCLLFTYSMLMYVSLPRLPRTWSLRVLTGWYWLYCILLTVIYRASLTAILANPE

>BdorIR75d Cluster-47430.0_m.43387

DFVAETYRVRAGIMFRQPPLSAVANIFAMPFASDVWIAILLLMIFTIGIFIVELVYSPHLHEMDILDCVVFVWGAMCQQGFYANLLNRSARVIIFTTFVSTLFLYTSFSANIVALLQSPSEAIQTLSDLTQSPLEVGVQDTQYNKIYFNESTDPVTNHLYHKKIAPKGENIFMRPSIGMEKMRTGLFAYQVELQAGYQIISNTFSEPEKCGLKELEPFQLPMIAVPTRKNFPYKELFRRQLRWQREVGLMNREELKWFPQKPKCEGGMGGFVSIGITECRYALGIFGFGLLLSAFSFILELVVNYVWNLAKKIHRNKKQRKESNAADGYHGNFVH

>BdorIR84a Cluster-5530.0_m.5124

MAVPKGATTARRQTFAAALLCRRTSLITTLILLLSKWNLCVSTVANAYEFDAFADVLKQQHLHHAIIAYNGDTEQAQQQAGLLKDNALRALLNVASLQFYDVHQAESAKNATDFQRLFYHDSPRVGIYVAQLEDVLLQQYVLGSNVISVDTIDAGGYRVRVDVGSRFNSSRVWFIMSKQRTVTAALANVRRVMTPLPLNISADITIGVRLDDNNTIQLFDIYKIQKDWLDIEPKGYWSPTEGLKLNLRFHQTFVNRRRNFKGLQLVGGIVIREQPADMADLDYLNSLCHKNFDPMQRKTYQLVKLMEPVFDVSFQPALRKTWGEQAPNGSWDGVMKLLLSGEAEFSLCPMRFVPNRVHLIHYTIAVHTEFVFFIFRHPHRNDIHNIFFEPFVEEVWYTVIAIVALTTLLLQLHLHHENRFFINKDPHFQTRFDYAIFSILEAFFQQGPSTDAFTATSTRTLIFSVCLFSLLLQQFYGAYIVGSLLSVSPRTITNLEALYNSSLDIGIENIPYNIDTFEKTTVPLGMAIYKERVCKNRERNILYIAEGAERIKKGGFAFHVSANRMYYILKELLTEKEFCDLQDVPFIPPYRIGIGITKSSPFREYFTTTIAKFHTTGLLQHNDNQWQLPQMDCSLSQNYEVEVDLQHFLPALLFLVSAMLLSLAVLILEIIYYNLEKSTKLARLCPRIMPKPKLEFIN

>BdorIR64a Cluster-52098.0_m.47708

SPIFFRIERAKVIDYTTRTWVARPCFIFRHPPSTKKDRIVFLQPFSNMVWILLGLCGIFTICLLWLLTSVERRLEAVGVVKQLGHSTRSSNMNIGGATTNNQQASAIGGNDELPPVGCRCSTRTTLSAGTMPSCGARNAPSVAAATKEQRKQKPGKLMQRSKTEARKNVQSISCRHCMHGCGSACCGQTGSDVAQQRVGLFFESMLFYVGSICQQGLTFSTSFFSGRCIVITSLLFAFAIYQFYSASIVGTLLMEKPKTIRTLRDLIHSSLAVGVEDIAYNRDYFLRTKDPIAIELYAKKVTSVPTDSETPSETTADNATALATLQPNVELTEAEKAKAYRDILHSHETGAHAKTNEASNWYDPAYGVKRIRRGKFAFHVDVATAYKIIADTFSEKEICDLTEIQLFPPQKMVSIVQKGSPLRKVITYGLRRVTESGLMDYQRKVWHSPKPRCVKQIHTDDLRVDLQTFASALLVLIFGCAVSLLALSIEIIQHKLWQRYRALEEDDDDVDDEETVE

>BdorIR75a Cluster-9086.0_m.8312

VDAEITYAFFNSLEADWNSTAYSSYTLYDVYNNGYYYGAKLNMTLDREIYCNDEGCFVNKYLSKLHLRNKYGNRNKLHDATLRLTVVVTKVPLTSTPEEIFAFLRSVNGTNKDAIARFGFQALSILVDVLGCKVNHTFVNRWTINETHGGLIGALAVQSADFISTPFIPTLPRMEFFTITAETSSFRSICLFRTPRNSGIQGDVFLKPFNTTVWTLFALLLLLTAVVLWSIFRLERYRMYKRYIDYMPSLLATFLISFGSACSQGSDMVPGSMGGRMVFYTLYLLTFLMYNYYTSIVVSSLLGSPVKSDIKTMGQLADSSLEVGLEPLPFTLTYLNNSLLPEVRRFKHKIDSVPNPKAIWMPLEKGILRVRDQPGFVFG

>BdorIR76b Cluster-20138.0_m.18336

MTGFDLILSAALCLTCANLTDIRLPEGLIELDENNTVVTISPDLAVDEPSLDDAPLETVKTIIAKKEKMDKLREWIKGRKLVIATLEDYPLSYTVMENDTRVGKGVAFELIDFLQEQMQFTYEVVVPEDNIIGSREDYEKSLIKMLNNSEADLAAAFIPTLSEQHSFVFYSTTTLDEGEWIMVMQRPRESATGSGLMAPFDFWVWILIFISLLAVGPIIYMLIILRNRLTGDKEQKPYSLGHCAWFVYGALMKQGSTLSPIADSTRLLFATWWIFITILTSFYTANLTAFLTLSKFTLPYNTVSDILYKNKHFVSARGGGVEYAIRNTNESLSMLTNMIRNNHAVFSSSSNDTFNLQNFVEKDGYVFVRDRPAINHVLYADYRYRKTISMNDEKLHCPFAMAKEPFLKKNRSFAYPLGSNLSELFDPKLLNLVESGIIKYLSTKDLPNAEIC

>BdorIR93a Cluster-20742.0_m.18916

MRFHAFLWSLWLPLCLALLPQNAAANDFSSFLTANASLAVVVDQEYMQRRGENVLASFQKILSDVIRENLKNGGIEVKYYSWSQIRLKKDFLAAMTVADCKSTWQLFDSTQQNSILLIAITDANCPRLPLNRAIMIPIVDEGQELSQIILDIKVQRLLRWKTAAVLLDQTILHDNPTLVESVVHESAKNHITPFSLLLYQIDDTLRSQKKRTAIRQMLSAFQDGGQTPRQFIVLSQFYEDIVEIAASMKLFHVYNQWVFFVLNEELRNHDPISVTQNLDEGANIAFALNTTESTCSSSINCTITELSLALVTSISRMIVEEQSIYGEISDEEWEAIRYTKQEKQDEMLGYMKEYLREYSKCTSCSHWKIETALTWGKSEEHRRYQSNSELRDTRNKNFEFIDVGYWTPTLGFNTHEVMFPHITHFFRNITLNILTVHSPPWQILERNSRGDIVRHSGISMEILKEMSRMLNFSYILHEVKVSAADSAEDMQHTNNVTDDLFGSLTFNIPYQVIETMQASRYFMAALAATIDEPDKKSFNYTVPISVQMYTFISRQPDEVSRIYLFAAPFTTEIWGCLVAIIIITAPVLYFINRLVPMDHLRITGLSTLNSCFWYIYGALLQQGGMYLPKADSGRLVIGVWWIVVIVLVTTYSGNLVAFLTFPQFQPGIDYFFQIFSSSAVQQFGLRNGSYFEKYATQITTRDDFRDYVQRATIYNNVQGEDIGAVQDGKRINVDWRINLQLIIQKQFEKDKECKFSLGRDNFVAEQIGLIVPRDSPYLQLINDKIMRMFQMGFIERWHQINLPSMDKCSGHGGMRQIMNHKVNLDDMQGCFMVLLFGFFIALFILFVEYWYRWYFVEKKRGVFAT

>BdorNMDAR1 Cluster-27519.0_m.24973

MPVLNGINVVYLLFCGIHLGVIAQKHSQHSDNPSTYNIGGVLADPESESHFRTIISNLNFDQQYVPRKVTYYDKTIRMDKNPIKTVFNVCDKLIEKRVYAVVVSHEQTSGDLSPAAVSYTSGFYQIPVIGISSRDAAFSDKNIHVSFLRTVPPYYHQADVWLEIMFHFGYTKVIIIHSSDTDGRAILGRFQTTSQTNYDDIDVRATVEMIVEFEPKLDSFTEHLIDMKTAQSRVYLLYASTEDAQVIFRDAAINNMTEAGHAWIVTEQALHANNTPVGVLGLVLEHANSDKEHIRDSVYVLASAIKEMMSNETITEAPKDCGDSGVNWESGKRLFQYLKTRNITGKTGQVAFDDNGDRIYAGYDVINIHEKQKKHVVGKFYYDPEKAKMRLRINDSEILWPGKQKKKPEGIMIPTHLKILTIEEKPFVYTRRLTDDEVNCDEDEIPCPLFNATDGSENENCCRGYCIDLLNALSHRINFTFALALSPDGQFGHFTLKNVSSSSSGAITSRKEWSGLIGELVNERADMAMPLTINPERAEFIEFSKPFKYQGITILEKKPSRSSTLVSFLQPFSNTLWILVMVSVHVVALVLYLLDRFSPFGRFKLSHTDSNEEKALNLSSAVWFAWGVLLNSGIGEGTPRSFSARVLGMFWAGFAMIIVASYTANLAAFLVLERPKTKLSGINDARLRNTMENLTCATVKGSSVDMYFRRQVELSNMYRTMEANNYDTAEQAIQDVKKGKLMAFIWDSSRLEYEASKDCELVTAGELFGRSGYGIGLQKGSPWTDAVTLAILEFHESGFMEALDKHWIFHGNAQQCELFEKTPNTLGLQNMAGVFILVAAGVAGGVGLIIVEVIYKKHQVKKQKRLDIARHAADKWRGTIEKRKTLRASLAMQRQYNVGLNATPGTISFAVDKRRYPRMGPRAPEQAWKSDADILRNRRYLDDATKGGHSPAVHMPILGKMRPPTNMLPPRYSPAYTSNVSHLVV

>BdorIR25a Cluster-19043.0_m.17308

MPRAYLKFYNNIVIFLKILSLVSLATGQTNQNINVFFINDADNEPAAKAVTVVSTYLKKNPSYGISIQIDQVEANKTDAKTLLESICSKYAESIDRKQPPHVVFDTTKSGISSETVKSFTQALGLPTISASYGQEGDLRQWRDMDESKQKYLLQVMPPADLIPEVVRSIVRKMNITNAAILYDDTFVMDHKYKSLLQNIQTRHVITGIAKEGKREREEQIEKLRNLDINNFFILGNLMSIRMVLESVKPTYFERNFAWHAITQSEGEVSSQRDNATIMFLKPMSYAQNRDRFGRLKTTFNLNEEPQIMSAFYFDLALRTFLAIKDMLQSGAWPKNMEYIGCDEFQGGNTPERNIDLRTAFTMIQEPTSYGVFELVTQPGKSFNGYSYMKFEMDINVLQIRGGNSVNTKSIGTWTAGLDSPLVVKDEDVMKNLTADTVYRIFTVVQAPFIIKDEKAPKGYKGYCIDLINEIADIVHFDYTIQEVEDGKFGNMDEKGEWNGIVKKLMDKQADIGLGSMHVMAEREIVIDFTVPYYDLVGITIMMQRPQVPSSLFKFLTVLETNVWLCILAAYFFTSFLMWIFDRWSPYSYQNNREKYKDDDEKREFNLKECLWFCMTSLTPQGGGEAPKNLSGRLVAATWWLFGFIIIASYTANLAAFLTVSRLDTPVESLDDLAKQYKILYAPLNGSSAMTYFQRMANIEQRFYEIWKDLSLNDSLTPLERSKLAVWDYPVSDKYTKMWQAMQEAQLPATLEEAVERVRNSTSATGFAFLGDATDIRYLVMTNCDLQIVGEEFSRKPYAIAVQQGSHLKDQFNNAILTLLNKRQLEKFKEKWWKNDETQAKCDKPEDQSDGISIHNIGGVFIVIFVGIGMACITLVFEYWWYKYRKNPRIVDVIEANSGGKDGKTIDSVILGQAGKEYDKGGNTVLRPRFHQYPTTFKPRF

>BdorIR8a Cluster-43369.0_m.39592|Cluster-39110.0_m.35527

HREWTFRNCGIMWLLQKLVILFTFGYVCANELKIAFWIDPLQADIELDVASTLKEIEALQLETKIQYYVVVITNVGKKKQEKNMEKLCEHLATDGVSVVIDFTYHIWREGLDLLRTYQIPFLRVDRMLAPYFKMFSEFVLQKSGHECIMIFQNARDTEEAIIQIVEGYPFRSLIMNAFDNKQDFIKRLRKIRPMPSCYAIFADGTAMNSIFDRISKANFFERPREWHFVYLDPRDRVFKFKKQVDYATKFTINPKTLCRALRMKDTYCLSGFSFQRAMILEILRGLIELKQANLNWLQSFVMECNATSPIENGTAGLDILEQFPMSEFLDLTTDVTFPNDEFEHVPRLTYTPTISINLYSSEHDAVTELAIWQNDNLRKINETISPPRRFFRIGTVEAIPWNYMKRDPKTDELILDSFGNPIWEGFCIDSIQKLSERLNFGYMLVPPTSGEFGRRDVVNDVWDGIVGDLVTGETDFAVTALKMYSEREEVIDYIAPYFEQTGISIVMRKPVRQTSLFKFMTVLRVEVWFSIIAALVGSALMIWLLDKYSPYSYRNNRAAYQYPCREFTLRESFWFALTSFTPQGGGEAPKAVSGRIMVAAYWLFVVLMLATFTANLAAFLTVERMQTPVQSLEQLARQSRINYTVVEGSSTHQYFINMKFAEDTLYRMWKELTLNVTEDFQRYRIWDYPIKEQYGTILLAINGSEPVKNAKEGFRKVNEHENADFAFIHDSSEIKYELTRNCNLTEVGEVFAEQPYAIAIQQGSHFADELSYALLELQKDRFFEDLKAKYWNMSRIKACSVNEEQEGISLESLGGVFIATLFGLGLAMVTLVLEIIYYRRKYSTMQRFSEITKVKPASGTSIKQLLPKKKSKKRIAVWHTSTSKRDNSPEHKTPPPAFDAVKFRGKKVPPNITLGGQVFKPGRAGQRQLSESLDSAEYRNEGIPNRDDELPPYTE

>BdorGluR7b Cluster-42653.0_m.38945

NVPIVNQKLLANVNWLVTIGTLMQQGSDINPKSLSTRIVSAIWWFFTLIIIASYTANLAAFLTVERMITPIENAEDLASQTEISYGTLESGSTMTFFRDSIIETYKKMWRNMENKKSIAFTSTYEEGIKRVNQGNFAFLMESTMLDYIVQRDCNLTQIGGLLDTKGYGIATPKGSPWRDKISLSILEFQEKGNIQMLYDRWWKKAGDTCLRKSNSKQTKANALGLDNIGGVFVVLFVGIGLAAWVAVFEFWYHYRSRRRASVYYENEHCTVERVMTEMTLNDFDSVYNSKKENVEEIQVKDQKLTCKHITAYCCKEPTAQRSLCGEMLDEFRYALRCMDSHRRPALKRSCPTCHILNDVKEFIDTESRSSMAVHGSLIEGTPKYL

>BdorGluR7a Cluster-36451.0_m.33169

TEPAVAYDSVYIFAIGLTSLQQSLTLSVSNASCASEIPWDGGLSLINYINSVEWRGLTGPIQFKEGRRVKFKLDLVKLRQHSLVKVGEWTPQTRLNITEPALFFDGGTINVTLVVITILETPYVMMHYGKNYTGNERFYGFCVDILELIARDVGFDYIIDLVPDRKYGAQDPFTGEWNGMVAQLMKYKADLAVGSMTITYARESVIDFTKPFMNLGISILFKVPSSPASRLFSFMNPLAYDVWLYVLAAYFLVSFTIYVVAKLSPIEWRDKHPCDIKNPIVTNQFTLANSFWFTIGTL

>BdorGluR4 Cluster-30699.0_m.27818

MKSLLLQIIYALLYAGAYAAEERPKFNVGIIFASKNDETEIAFRTAIERANVFERSFELEPIVEYADTDDSFMVEKTVCKLIAQGVIAIFGPNTAGGTDVVASICNTLDIPHIVFDWTPSEALSDRQHSSMTLNVHPNNILLSRGLAEILQSFSWRSYTIVYETERELQQLQDVLQVGEPSSNPTTVRQLTEGPDFRPFLKNIKLSTDSCIVLHCSTDNVMKILKQANELRMLGEYQSVFITVLDTHTLDFQELLSVNANITTIRIMDPTDYQVKNVAHDWEEHEKREGHYYRADPSQVKTNMILANDAVSMFVKGLAELGIAEELNPPKLECRKNRAWTHGRRIIEFLKARSVEAATGRVDFNEHGERNFFTLRFMELTTSGFLDLATWDPVNGVDSLEKEDASEKRVGEKLANKTMIITSRIGAPFLLNREPKDGEILQGNARYEGYSMDLIDAIAKLLNFKYEFVLAPDGKYGSFNKLTQSWDGLVKQLLDGNADLGICDLTMTSARRQAVDFTPPFMTLGISILYAKPEQPPPDLFSFLSPFSLDVWLYMATAYLGVSLLIFGLSRMAPADWENPHPCKEPEEVENPWCMSNTTWLAVGSIMGQGCDILPKAASTRLVTGMWWFFALMMLNSYTANLAAFLTMSRMESSIESAEDLAAQSKIKYGALLGGSTMGFFRDSNFSTYQRMWTAMETARPSVFTKNNDEGVDRVLKGKGLYAFLMESTTLEYIIERNCDLMQVGGWLDYKTYGIAMPFNSPYRKQISGAVLKLGESGMLSELKRKWWKEMHGGGSCSQAESSGGDTPELDLENVGGVFLVLGIGLLTAILIGMCEFLWNIKAVAIEEKISLSEAFKAELMFALRFWIQTKPVNIASSSGGSSSSSSSSSKSSKSSKSSSSSSSSSTKRSKRSSKSYARSISQSTKSIINVTHDLEKNDLSVHDKLRKISSMFSLKSAPSEPSVANVHLDPPPALKHNSQPNHHHAVDKHTQMVREVAQQTTLTSDDGSDRDEVEAQQPEIDEIPIAQPHQHHNHNHHHHHHHHHHKNHDHNHHPQLDDHQPAMQLVE

>BdorGluR5

MVFLQEFTKCDVVFITTPNLKTKDNMETINLKQIRLNSNPKLNNCVCFNLRSFIGYESLLSRCKLSVSHILLIYYLCNFLCAASLPAVIPLGAIFTEDQRDSSIEYAFKYAVYRINKDKLLLSNTQLIYDIEYAARDDSFRTTKKICRQLESGVHVIFGPSDALLSDHVQSICTSFGIPHIESRIDIDENSKELSINLYPSQRLMNLAHRDLMIFLNWTKIAVLYEDNLGIFNHQDLLHVTADIRTELYIRQTSPKTYRQVLRAIRMKDIYKIIVDTNPKTINAFFRAILQLQMNDHRYHYMFTTFDIEIFDLEDFKYNGVNITAFRLVDVESQRYKEVIEQMQKLPHSGLDYINEKPYIQAQSALMFDSVYSVAAGLMELDKKDLLSWHNISCKNELPWRDGMSLYSYINSASMNGLTGRVHFTEGRRNLFQIDLLKLKREKIQKVGFWKPEVGVNITDSTAFYDTYSSNTTLIVMTREEKPYVMVKSGISQTGNDRFEGFCIDLLKAIATQVGFQYKIELVPDNMYGVFNPDTKVWNGIVRELMEKRADLAVASMTINYVRESVIDFTKPFMNLGIGILFKVPTSQPTRLFSFMNPLAMEIWLYVLTAYILVSLTLFVMARFSPYEWNNPHPCMKESDIVENQFSVSNSFWFITGTFLRQGSGLNPKAVSTRIVGAIWWFFTLIIISSYTANLAAFLTVERMITPIEGASDLAEQSDISYGTLEGGSTMTFFRDSKIDTYQRMWQYMETRHSAVFVKTYEEGIKRVIEGNYAFLMESTMLDYAVQRDCNLTQIGGLLDSKGYGIATPKGSLWRDPMSLAILELQEKGIIQILYDKWWKNTGDVCNRDEKSKESKANALGVENIGGVFVVLLCGLALAVVVAILEFCWNSRKTLQLTETQTLCSEMTEELRYATHCHESKQRQSLKRNSLKFPPDSTYVPADSGSGILNSSGVHYNYFD

>BdorGluR1 Cluster-44640.0_m.40769

IVAIFVISYLLVISARIAEDEWENPHPCNKDPDMLENKWDLFNTFYLSAASIMQAGCDMLPKSAPFRTFTAMWWIIAVIIPNCYTANLAAFLTSSKMESTVQDLKGLVEQVDIKFGTIEGGSTYTLFAESNETVYRLAYNMMKNEDPSVFTKDNKEGVDRVLKNNGSYMFLMETTALEYNIERSCHLRSVGDKFGEKHYAIAVPFGAEYRYNLSVNILKLSETGKLFQLKNHWWKVNDTDCEDNDDDADNDSLGIYEVRGIFYTLYLGLLAAYLMGFIEFLMHCHSRASEEGLRFKEILVNEMRFVLRIWNNRKPVSCTPTASIAASSRRSSNRTARTLTKKGSQQSSGSGEELKELANNKVKKNGTIIKVDEM

>BdorOr10 Cluster-21927.0_m.19981

MFDDLQLIHMSVRILRFWSLIYEHTWRRYVCLSMTTFLVFTQLYYMFRTSEGIDSIIRNSYMLVLWFNTILRAYLLLYDREKYEKLLSDLETFYYDLKRSKDSYIQDLLVEVNTTGKYMARGNLFLGLLTCFGFGFYPLFATERVLPFGSMIPGVEEYKSPFYEFWYIYQMVITPMGCCMYIPYTSLIVAFIMFGIVMCKALQFRLKTLHRVRHIESLIHKNVRECIRYQLSIIDYIARVNALTTYIFLLEFLAFGTLLCALLFLLIIVDSSAQAIIVCAYIAMIFAQILSLYWYANELREQNLAIAAAAYDTEWFTFPIPVQKYILLMILRAQKPPAIMVGNTQPISLELFQSLLNASYTYFTLLKRVYT

>BdorOr7 Cluster-3194.0_m.2920|Cluster-49744.0_m.45516|Cluster-1732.0_m.1516|Cluster-13769.0_m.12515

MVTAVVDNPMLSVNVKLWQFLSVLFARDWRRCVALVAPVCLMNAMQFVYLYQQWGDLSTFILNTFFAVSVFNALLRTCLIIKNRDKFEALMEELVTLYDDIQDSGDDYAKSVLAAATKSARNISIFNLSASFSDLIVAMAYPLFQQQRVHPFGVALPGIDVTRSPLYELIYIGQLSFPFTLSSMYMPYVSSFATFSMFGKAALQILQNNLRNLCDNMKSKTEEQLFEILRKNIAYHARIARYVSDFNELVTYMVLIEFLLFSCVICSLLFCINITTSTAEKISIVMYIGTMLYVLFTYYWQANGVLEMSLLVSDAAYEMQWYNCSPHFKRTLLIFIARTQNPLQIRVGQMHPMTMEVFQSLLNNAYSYFTLLHNLYND

>BdorOr17 Cluster-8386.0_m.7721

MAVKKWSPRNTSSMSRTASANIIIAVLKSLGYWQWTRDPRQPYIEKVERAYRIVLHTTLPFTFIALMLTGVLLSRDLDEIGSILHVLLTEFSLIVKTLHIWRKGGVAWRFMHEVANDPIYDLRQQSEWTKWQQAQRSFAIVSNTYFVAATTVVVFACIGAMMTPADVYVLPMNIYVPFDWHHPRRYWYAWTYNTIASLMTATANAMLDLVNCYFMFHLSLLYKLIGWRLSALRRSANEPPVIEQMSEIFQMHMKVRRLTTECETLVSIPVFSQIILSSFILCFCGYRLQQMEIMENLSMLFSTVEFATVMAVQIFLPCYFGNKVTESSDALTDEIFNSDWTTFDVPTRRFMILYMELLKKPANLMSVNYFIIGVDIFAKTMKNAYSIFALVLNMNN

>BdorOr5 Cluster-46823.0_m.42801|Cluster-54166.0_m.49567

MTLKINSWDAFKYHWRVWDLSGFRGPQRQSVWYIPYKLYTIAITLLFPIYYPICFTVESFLADNLNDFCEVIYIAMADMTLNIKFLTLFIVRRQLLELRPILKRLDARAKTEEEMNVLQEGIDSAKKCFLIILRLFYSAFVTSQLMVIFSAEARLMYPAWYPFDYQASRTKFWIAYGYQTIGFLVQCTQACSVDTYPQAYMRVLTAHIRALSLRIERIGRQNFSGVSSELMCSKENEMKRNYEELVSCIKDHKTIIELFSTIQKPISGTSMAQFVCTGVAQCTIGVYMLYVGFNISIMLNMAVFFVSVTMETLILCYYGDLFCQECEELSKAIYNCNWTVQSSEFKKVLCFFLFRSQRVNVLMAGNWIPVRLPTFVMVVKSSYSIFTLLSSFK

>BdorOr2j Cluster-9369.0_m.8539|Cluster-41584.0_m.37912

MFDLIKGRGRTVFASRDAVIYLFNSFRYLGINPPAKYRLPYFMYSAIITFFAVLFSPVIFNVGWLRDRNKLSVMEILTCVQASLNVMAVPLKCITLAMAQKRLRGIEPMVTELDERFPTQEDKAKIKKCAVTGNQLVFGFAVSYFMYETLTVVSALVGGHAPLSLWIPNVDWHRSTWEYWLQVSFDAAVLFFLLYHQVLNDSYPAVYIYIIRTQVQLLTSRVEKLGYDEQKSVDENYQELLECIVIHQKILKIVKIVESVVSITVFTQFLVAAAILGVTMINIFIFADLTTKIASVTYFFCVLLQTSPTCYHASYLLDDCDQLRIAIFQCNWIAQNKRFNNLLIYFLHRSQDSMPFFALKLVPINLATNLSIAKFSFTLFTFIQEMGLGENLKG

>BdorOr2h Cluster-48353.0_m.44293

TVQFLSGLQASLNLIGLPVKCLTVTSALNRLRGMEPTLAALDARYTRPEDMTLIRKAAVMGNRLVFFFGTSYLMYMLFTVIPPLINGKAPLSVWIPFYDEHQSTMHFFGQIVYDLFLMGFVLFHQVLYDSYGSVYIYVISTHLQLLVRRVGRLGTDATKSKDDNLNELVDCVVTHQQILELLATIEPIISKTIFTQFLIISSILCVTMVNMFFFADRSTQIASTLYFLCVLLQTSPCCYFATELKADSEKLPLAIFHCNWPEQDRRFRKVILYFMHHAQLSIELMAMQLFPINVATNISLAKFSFTLFTFIKEMGIGQEA

>BdorOr2g Cluster-46793.0_m.42767

MSKILRVRSATVYKSRDALTYLFNVFTFVGTNPLENRSQRYYRIYYFYSFTVNFICCLFCPLSFHIGYIKLRHVLTNSQLLAAIQNAVQVSGIPIKILVITWYMKRLRHAFEILDELDVNYTRREDLAKIRECVRRCKKIVLIFCFPYYSFELTTIALGVAQNRAPLAAWVPFLDGQRAAWEYWTIVLWDAFVMFFLLCHQLGSDTYPPIFINIIRTHVQLLIARVNRLGRAGALTADEHYEELLGCIRTHVQIVSIAKIVAPVISVTLFTQFATTATTLLNWLGNVEYPENIISLAFFSCQLLQILPCCSSASQLIADCERLPDAIFHCNWVDQDRRFRRAMLFFLQRAQNPIRFSCLKLFNVKL

>BdorOr2c Cluster-19396.0_m.17645|Cluster-15989.0_m.14544

QATEGSIEMRKIADLFYGRGKHDFETTESFVLLSRSFAAIGFLPKIPKRIVDVIHQLICWSCIFSCPYLFVSGVVKTMHSLPITIVLAHLGVAINSIVFPLKAVYIKANIDRVDDIGKIFNALDKRYQRPQDQMQIRDSVKTCTRIFVVFCIVYWLFGISSWLVALCIHEYPHGNNLPFIDWLPESNLRFWLHFIFEVVFLHQLLQMSLTMDSLPALYIHALRTHMNLLTDRVSRLGLNPDFSDQENFEELVDCIVSHREILQISDTVGKILSLTTFFQFTVYAAILCVCMLNMFVFGDASTKLVTLVYLLPVFGQTTPTCYQASMLEADSAKLPLAIFHCNWLALDKRCHKLIIYFMQRAQQEISFTAIQLFVINLRTNLSIAKFSFTLYTFINEMGFGETLKDRLE

>BdorOr2i Cluster-52111.0_m.47721|Cluster-47713.0_m.43662

DIIHQIICWCSILTCPVWYFAGLIDMMDDLPITLLLSNLGVAINCIALPLKAIYIKVNMNHLHDINLLFKRLDERYQTSEENIQIRESVKTSTRIFAACCILYWFFGISSGLVPLFAHEYPHGNVFPFIDWLPEGNFQYWLHSIVEIINLQYLLHLQSINDSFPAVYIRNIRTHIRLLTNRVSRLGLDPDLSDQQNFEELVDCIVSHQEILVISDTVGSILSLTTFFQFTVYAALICVCMLNMFIFGDLKVKVSTLIYLIPVIWQTVPTCYQASMLETDCSKLPEAIFHCNWLDLDKRCHKMIIYFMQRTQEEICFTAIKLFQINLGTNLSIAKFSFTLYTFIKEMGLDAHYNQK

>BdorOr2b Cluster-14614.0_m.13277

WELTGIKLLHPYKFLNLLHIVFSWILIVLSPISMGTVIFNTMGDTSMTVTLTSLQASLNALLLPFRTLVIAINFKRLRSVEQIFENLDQFYVREEERLEIKNCEKTCRRLFFTIFILYNIYGVSTWLVALFAHNRDMFWLIFIDWIPYQPLRHCLHFLSEVVMIHFLLQASVSSDTFPAIYIRALR

>BdorOr2f Cluster-42288.0_m.38575|Cluster-6576.0_m.6029

MRSFFDLYYGRGSEEFETNESFQLILWCWALIGVKPLKPYGFFRFLQMTFCWFCLFMGPVIFTVGFIQVTKQTSSMTVILTTLQGSLNSLGLPLKAFVTVFYLDRLRSVEPIYKSLDARYQNPQARFAIRNNVIQSTHLFVTLVVSYFTYGTISWLSSAFTHTQTANIWLPFVDWIPHPTIHFWLHFVIEVVYLHYLLIAQCMNDLYPALYIKALRTHITLLADRVSRLGENPELTDEDNYQELSDCVRSHQELLKISRAVGSVISITLFIQFTIAATVLCVCMLNLFLFADASHRVIT

>BdorOr2d Cluster-27290.0_m.24778|Cluster-13145.0_m.11980

MQRFSDFIYGRVESDCETNKPFKVLLAFYGLIGLKAKPRGFLPTLHMVFFCIAYAYTPFLAIVGFLRFQKTATVTESLSALQAFINAIFAAAKSVAVLVNFKRFQSVEPIMKSLDERYKTPQERQQITDCVADCTRLYAAMGFIYYLYGLISILTALIIHKQPFGGWYPFLDWISNPTVHFYSCVAFETWYLYFLLTAQYLHDVYPTLYMRTIRAHMQLLRERIGRIGVDPEKSVDENNKELIDCIATHQQILQVVDMVRSVCSPTIFMQFVCVALVHCVCMVNIFIFADTLNMVITMWYYLMVATQILPTCYEASTLEMESSKLPVAIFHCNWLALDKRGRKLILFFIHHAQKEVTFVAMQLFDINMRTYLSIAKFSFTLYTFANEMRFGQNIKESVE

>BdorOr2e Cluster-31943.0_m.28967|Cluster-16474.0_m.14968

MRKLTDLLYGRGVTKFETNESFQLLIQCWSLIGVKSLKPYRFGRLLHMCFCWFLLLLCPLTFFMGYVHTLTTEPITIQLSLLQTAANVLGVPLKTISVAILRTHLRKVEPIFDRLDERYQSVAGREQIKDCVMTSSRIFASVGFMFHFYGSTTYLQALLTRGYPMRAWLPFIDYIPQPTIRYWVHFIFEVFHMASLLTLQASMDAFPAVYIRTLRTHLNLLTERVSHLGENPEFTDEENYEELVDCIVTHQELLEAKNILGSVCSLTLFIQFVIVALALCVSMLNFFVFADRQQQVVTVTYYLGVIVQIMPTCYQASMIEADSAKLPDAIFHCNWLAMDKRSRKLIIYFIHRAQKNITFVALKFFNINLTTNLSIVKFAFSLYTWMSNMGFGDNFKDLLE

>BdorOr2a Cluster-1293.0_m.1167

SRLGTNSVFNDEQNFRELVACISSHQQILQVADIVGSILSLTVFLQLAFAAAILCVCMLNIFIFADTMHKVTTIVCYLVVLMQTVPSCYQASMLEADCAKLPDAIFHCNWLDMDKRFRKLIIYFLHHTQTEITFTGLKLFRINLSTNLSIAKFSFTLYTFMNEMGFGKNVKERLE

>BdorOr26 Cluster-7854.0_m.7179|Cluster-37633.0_m.34241

SASTFLASVSSGYPPYSLYFPFLKWRRSRTEFIIASLLEFIIMDFACLQQTVNDGYPVIYINMLRCHMKILQFRVEKLGTNPTLTQVEHLSELKLCIKDHQLLIELYDTIAPIISITLFIQFALSAVCIGTALINIVIFANEFQTQVACSFFILAVLIEIYPACYFSQCLINESDKLADVIFHSNWIEQSPEYRKLIIFFLQRSQRPMFLTAGKLFPVTLSSFVSIAKFSFSLYTFIEKMNLKERFGIE

>BdorOr12a Cluster-22861.0_m.20846|Cluster-29600.0_m.26899|Cluster-28161.0_m.25562

MILENKEELYKRNYNSIKVLFRVSYTLGVNLTAPDKFKDSLKVIQIILIASSLLSLFAHWWYLKRHIDNIPLIAEAVFTALQIGMAAIKLIYFFFTHRTFYRLLDQTLTHEIIRKIEILTDFPIDRQLRQEIDDIMNRVWRNTRRLFLFYFCCCVGIIANYFFTAFFVNLYHQLKQTPDYEFFLPVPALYPFWEKKGMTFPYYPIQMYMTGAALYVSGLGAVSFEGVFMVLCQHAVALVKVHNLLVQHATSPQIPAERRLEYLRYLIITYRRVSKYLQEIKTIFKHISLVQFLLSLIVMGFVLFEISYGLEASIVIFIRMIMYISASISQITIYCYHGQALTSACEEIPLAYYNCDWYGENKTFKNLILMMIMRTNKEFNMEVSWFTLMNLTTLISLLRASGSYFLLLQNLQED

>BdorOr12b Cluster-38619.0_m.35060

EFISEVNQLFRHICLSQFLHGLAIYGFVLFEMNFGLESNKITFIRMLMYLCAATSCDCMHYVNGQFLANEVIVKYPQCRPTEKMFICFQLEKVPLACYSCEWYHETDAFKKTLKMIIMRSNKEFYFQISWFTVMSLATLMGIFKASGSYFILLQDIDEP

>BdorOr12c Cluster-47467.0_m.43423|Cluster-3569.0_m.3311

MYNAAEFQELKNNNRFKIRELRKVSYILGINYGSETSLKRFLRVLNLFLIIICAISLYPRWLMLERADGNVPLIAETITTMLQTTTSMVKMTFCLFMQGQCRALLKKAENYELLQGIKIFLTDMDIKAELKVEINAIMATIWKESRRQLLSCLITCSCILSNYFLYAFFTNLYHQIKKTPNYVHILPFTGYPMFLDKGMASPYYAVEMFIGGCSLLTCGMCSVSFHCIFMILCKHACGLVKVLCVLLMRSTSLQVPAHRRDEYLRYCVIQHQQTLRFINDINDLFKHITLSHFLHSLAIYGLVLFEMNFGLETDKTTFVRMLMYIGAALTVDSMYYVNGQFLATELEKIPFVCYSCDWFNESEDFKRTLKMIIMRSNKDFCFQISWFGIMSLTTLMGILKASFSYFLILRDMTDETN

>BdorOrOrco Cluster-42752.0_m.39041

MQPSKYVGLVADLMPNIRLMKYSGLFMHNFTGGSGLFKKIYSSVHLVLVLVQFLLILVNLALNAEEVNELSGNTITVLFFTHSITKFIYLAVSQKNFYRTLNIWNQVNSHPLFAESDARYHAIALAKMRKLFTLVMLTTVASAVAWTTITFFGESVKFAFEKETNSTITVEIPRLPIKSFYPWNAGAGMFYIISFAFQCYYLLFSMVHANLCDVLFCSWLIFACEQLQHLKGIMKPLMELSASLDTYRPNSAALFRSLSANSKSELINNEEKEPTDLDISGVYSSKADWGAQFRAPSTLQTFNGMNGTNPNGLTRKQEMMVRSAIKYWVERHKHVVRLVAAIGDTYGGALLLHMLTSTIMLTLLAYQATKITGVNVYAFTTIGYLGYALAQVFHFCIFGNRLIEESSSVMEAAYSCHWYDGSEEAKTFVQIVCQQCQKAMSISGAKFFTVSLDLFASVLGAVVTYFMVLVQLK

>BdorOr4 Cluster-1434.0_m.1270|Cluster-8762.0_m.8041

VRRHKKRIADKNSRVFKMLFNPKPPKDPKNFRFPLQCIWLKLNGSWPLKPKVAGEFEKYFRLLYSTWAWYVVAMVGITIGFQSAFLLKSFGNIMVTTENGCTTFMGVLNFVRLLHLRLHQRDFQQLLAQFVKDIWITSSSHPTVERACARNMRVFQVISVLQSSLITMYCILPLVELYMLTLNVEPDVLDSMPKPFPYKMLFPYDANHGWRYALTYLFTAWAGVCVVTTLFAEDSLFGFFVSYTCGQFRILHTQIDNIIPDSYAATRAGRGTEVVFQRECIRRLDKIANKHCVLFNFVSRMEEFFSPILLVNFLISSVLICMVGFQLVTGQNMFIGDYVKFLVYILSSLSQLFVLCWNGDNIIQNVCIEYNN

>BdorOr6a Cluster-33869.0_m.30703|Cluster-10749.0_m.9794

LSRQRALRKLLRNFYRDIYFTPADDAALYKEINSIMRFMNIFTQFYYVPMMLILVLYVYDVASVGLASPDKPFIYRMSFRWYDAQVPLQFIITAIYSGWLTISCVTIWTAEDYTLCLVLCHASFRYKKLRLDLQQLLEMARADLKCGETPCTNQNLHIAFRRRLREIFRRQQRLNGFVAEAKAHFTHQIFYIMSFGVLLLCVVSFQFQSGPITVASSKYISWLISQTAQFLLIGYFGQMLMDETTELRNSFYCCRWEDLLVLGDPHSNKLLLGDVQFAIMNSQEPIVFDGMKFFPLTYSTVSAALRSAVSYFMFLNTMNGEN

>BdorOr6b Cluster-3731.0_m.3449

HYETDGDSGHIKSTRKLLRLVVSIMRYLPISYHKPLLPNGLHPPIDWQLYGFFCANGWPLAAHITKTRYIADIMVTIMQFMSESMVLIGEAVVMHDNLDNISFVCTVLAPNLILFEMMLRAYNIIYRRNSFRTHIEEFYKKIYIQRTWNPELFEKIRRQQLPTKYSTFTYIITLVTYVYVPVSGLIKNERLVPFPINFGFDYTVPWPRYLVFLTMSMWTGFAVVGPLVAEANILAMQILHLNGRYSLLLEDLRNISRKSIAEHEKCKRKDNMLVTQRFRYRLYDIIRRNVELNDFAKSMQEQYSFRVFVMLALSATLLCVLGFLTATLGITAQNIRFVSWIIGKVVELLIFGRLGTTLSTTTDKLSTSYYCCDWEDIILHSTNAEENKKLMKLIALAIHLNSNPFRLTGLNFSVVNYETVVAILRGAGSYFTVIYAYR

>BdorOr15 Cluster-18285.0_m.16650

LLFMVSAQLPMMNYIIYHIDDLALATACLSIVFTNVLTVIKTSTFLTYKREFKSLMAEFESMYDELQEAGAKRCLVTVNVGAKRFVKLYFSACTSTGLYFTINPLVSMIWAKFQAKPIPLELPMPMRFPFDFESTPGYEFAYIYTVFITIVVVMHATSVDGLFVSFTTNLRGHFQALQYFIETNTFDKSEALLQRELGIYVQYHVRLLGLAQSVQRIFKPIIFGQFLMTSLQVCVIIYQLVMNMGVIMEMVVYCTFLSSILLQLLIYCYGAEFLKTESSAVSTAIQMSQWYNLPPRHRHVLRLMMLRSQREIIISAGFYEASLANFMSILKAAMSYITFIQSIE

>BdorOr1 Cluster-2425.0_m.2142

MFKSDLGITGYFHLQKFTFHRLGIDMTTRSARVIKIYFLTLQIIALATILIPIAVYSWQHIQEIVEVTNAMAPFMQATISLWKIWRVIYRRKEMAQMAENIYLISTRASAKELTHLIQENNRERLMNTAYYYSVLNTGMLALAAPVLVSFIQYLRLGEFSYIVVLKATYPIDYARPLNYFLIWLWTAVAIYGVIYGSVSVDSLYSWYIHNLVGNFKILQSKLVTAETTSELSERRELIYYCIAYHQRIIAMTEQLNIIYQPIVFVQFSLNALQICFLAYQIGSGVVDTVDLPFLFLFMISVGIQLMIYCYGGQHLQNESVNVSKSIYQTINSSSWPNELRKVLLISMMRAQKPSKLTGIFFDVDLPLFLWVWRTAGSYVTLLRSVDQKTM

>BdorOr3 Cluster-25807.0_m.23454

MNFRFLSRTFPLRDYYFYVPKLCLGALGFWPLDTSAPNASNVWAWVNLIILTIGVFTEIHAGCTVLKTDLELALDTLCPAGTSAVTLLKMALIYYYRKDLAWVLKRMRDLVYERDVSINTVKKHIVRAHAVMAARLNFIPFVMGFITCTSYNLKPLLMTLILYMQGQEPMWKLPFNMTMPSFLLHAPYFPLTYIFTAYTGYITIFMYGGCDAFYFEFCSNTAALLELLQNDLKSIIKFDQLSLTTEESTVLEWRLVQFIKRHNDIIELTRFFCKRYTVITLAHFVSAGLVIGASIFDLMTFTGFGIVIYIGYTIAVLGQLFIYCYGGSMVAESSVQLATVAFGCDWHACNPRLRRYVLMIIMRSQRAISMSVPFFAPSLITFTSILQTSGSIIALASSFK

>BdorOr24 Cluster-38138.0_m.34693

HLNIIKSTFDDLILDECHMRRDMKRIRDPNSRMADIVEHHCILKSVRDDVEHIFRLSILLQFFTSLVISAVTGFQATMNASNSNSEMIIYFYCFCIFTQLFGYCWFGNEVNEQNKTLAAHGYGSSWYHFDQRFRKSLAIFLLNAQQPFNFTGGGFVDLSLPSFTNVLSKAYSFIAVLRQMYER

>BdorOr18 Cluster-14233.0_m.12979|Cluster-48777.0_m.44673

SLTHIAGVFKVINIIYRLDEVAFVVRRIEYAAKTYVISKSQLVAFYRGEFENKIPLTIYASLVGFTGVFGLIYLLYNPIGVAGQIFPYRVKLPEWMPFGIQLAYMGMSVLVFALQIVAIDYLNVTMINQIRFQLKILNLAFEELKLDCVNARELEEVNKRLHTIVEHHCLLHDLRNDVEDIFRLPVLLQFFTSLIIFAMTGFQAIVKVENSNGAALIYCYCGCIFCELFVYCWFGNEVSEQSKTLAASGYGSHWYAFDQRFKKSLLIFMCNSQTPFVFTAGGFMSLSLPSFTGILSKSYTVIALLRQVYSR

>BdorOr23 Cluster-35957.0_m.32725

FLSYKATYRYKAFSTHSEMTTTKVRPTESFGKIIKFFHLISSLVGADVADENYRVNIITITLIICIVAYFIFTGTTVASVFSENWTYLLEASCMVGSVLQGITKLISAFAFAKEILGIRIELENLYREYEVKGDDYAEALNKSCERVWQVIKMVGQVYFVAGGGIILITIVLIFASNEKVFLMHFMIPGIDVDTQVGYLMTLTLHTMCFLFGAFGLFAGDLFFLLFLGQPMLFLDLLVLKVKSLNEAAAENSSNAERLLIEIIEWHQYYTDYNLRCNRIFYYINSMQIVTSGISIICTLYIILLGDWPGAYLYILVAFGGLYLYCIMGTKIQTCNTAFCEELWNINFYDLEVKNQKMIIPILMKAQNPSEIKVGGFLPLSVQTALQITKTIYGIFTMMLRFLEENQ

>BdorOr22 Cluster-33833.0_m.30674

SKFLFYHGREMSIKNIRPTASFAKLVKTVRFISSLVGADVSTVNYQVNIITIIVIICIIMYFIFTATTVASVFSENWTYLLEASCMLGSVLQGITKLISGISRTNEVSGMRLELEELYRVYETKGESYCKVMNACCERVWQLIKMVGLIYGAAIVGNLLLTSFMLFFTNQKIYIMHFFIPGVDVETSFGYLLTTALHSLCFLAGCFGLFGGDLFFLIYLGQPELFRDILILKVHELNEAAAQKDNKTESLLISIIEWHQYYTDYNERCNEIFYYIITMQILTSGVSIVFTMYIILMGDWPGAYLYILIALSSLYLYCIIGTNIQTCNETFFEELYNINWYELDVKERKLMILVLMKSQNPSEIKIGGVLPLSVQTALQITKTIYGIFTMMLGFLDEEQ

>BdorOr13c Cluster-28293.0_m.25666

MKTQNRPSDIFYKMLSFIRFCSRQIGVDIIEEHYKINLNTYFVIAAIIIYCLCSVNTIRKYIATDWTVLLDVSSPISCTIQGLVKLMSALFYPKLYRKLTLDIGKIYEKYQEMGREYEEKLHTWNKSMKKLLIGCAIVYFVTGLLLLFTPVVMYVLKGERHLIWLCEVPGFERDSLYGYWVNNAFSVICVFIGCFGLYAGDVYLIIFLTHSMFFYDILALKINDLHKLIEEDNHADRQTELVNDIVEWHQYYLDFNDKCNLLFFWTISAHIICTTAGILSTLLIIMLKDWPGAYTYLLVCFLWLYMYCILGTRVEISNDQFCTGIYDIHWYALDVYNQKTIRLMLTQSQAPRNITIAGIEPLSVNTALKITRSIYSLAMMVLRFQTK

>BdorOr13b Cluster-26376.0_m.23992

MKTQKQPSDMYYKLLSVIRFCSRTIGVDITAEDYKINSNTYIVIGAIIAYYFCAINMIARYIRTDLTVLLDVFSPVSCTTQGAVKLMSALLYPKLYRKLATDIGQIYEKYQQMGREYEQKLLEWNKSMKKILFACAIVYSITAMLILSTPIVMYILKGERHLILLCEVPGFAADSYYGYWVNNAFNLLCVMIAAFGLYAGDLYLLLFLTHSIFFYDILVLKINDLHKLLEHEDKEDRQTRIVKDIVEWHQFYLDFNDTCNLLFFWTISAHIICTTTGILSTLLIIMLKDWPGAYTYLFVCFLWLYMYCILGTR

>BdorOr13a Cluster-23745.0_m.21669

RILDVADISLSKIYCNMQTKTRPSDNFCKLLKIIRLSSSLVGIDVIDENFKFNYVVGFVLVAIAWNFTISIYTIWKDVKTDWTVLLDVFSPISCATQGVVKIISILLYPKLYRELAMDLVNIYKKYEALGTKYETKLFEWNQSMKNILIIGGLVYFVSAVLALVTPIFLYIFKGERHLIIMCQMPYVDLETDHGYFITIGYNILCVFVAAFGLYGADLYVFLFLTHSIFFYDIFALKVEDLHEVLHENKQDTRIKAMVNDIAGWHQYYLDFNDKCNQIFFWTITSHILCTILGILTTLLIIMLKYWPGAYPYIFVCFVWLYMYSILGTRVEIC

>BdorOr16a Cluster-44840.0_m.40960

LCMYYEYLGKLLAEMNVQDAMDPTKSDAVYKQLHDYIYMHQYLNNLAVQLNDLFNFSILSSDAGIAISICFNVVLITEAKNNLQIINYTIPLFVEVWLIYDASKWGQMLETVTA

>BdorOr16b Cluster-18136.0_m.16515

IGLVLTNRGLMLGLNRAKLLKMYNAIDRIFPRSEHLQQHMEVEKVHNYIKKRFFYFHLFLTITACGFIFMPFVKFMAFHGFKSDAPVGEEYHVNASWLPFGVKDKVSTYPYIYVYEMFLATAAAHMLVVWDQSFVILI

>BdorOr14b Cluster-27558.0_m.25009|Cluster-24215.0_m.22065

DLAATEPFTWSGARTYSYRRIWLRRALFTFGAINLVYQNIGMLIYLFMPHESSAQSTIVQVTETGGIMGLTMVGTSNMLVMFWYGDRIAMLLEKFQQLFPTARLQRKAKFTKQSLRGVEFPHRIEHFVLKSNKLMKLATTLYMFAFAYYNSLPIVEFLYEWTTPGIVWKYRYQSNTWYPWQNERNAKSFASFTLAYVCQVQSSLTGVAFIMAAEFMLCFFTTQLQIHFDYLANALETIDAAGANANEDLKYLINYHSQLLSYSKETNAIFNVSFMVNLCTSAIAICLMGFSMVMISLAHAFKYSIGLTSFIVFTFFICYTGKELTETSDKLLNAAFYGNWYDGNLAYRKMILFFIMRCRIPTELRAYKFTTVSMPTFTAILRSSYSLFTFFQAMGQ

>BdorOr14a Cluster-22423.0_m.20477

FPSVAKQRRIAELNESEKGQIGSVGIYRLRYYEEKSRASMRRLTRFIINCYIYYNSVPILQLCYAIVTHQEKIMYRAQANTWYPWHNHNDHSSFIGFMAAFLTQAIAEYVCIVFIVCSENLFCFFTTQMLMHFNYLSSALSALDASAPDALLQLKALISYHNQLLRLVVRINSIFNLTFTLDLIITTFAISLMGLTMVLVNLTSAVMFSAGFSFFLILGYLFCKNGDELIRETMKISPAIFYSNWYEGS

>BdorOr19 Cluster-19325.0_m.17585

MNAIERNTNFTRFTAGPVRYFKFLGILLQQPEMPHSKYQRLLIVVTIALMFLHQIGYILEPGRTFAEQSAAAGLLNYTTVSGGKILFLVYNRRLLLSNHCQLAALYPSAAVERHYKLEHYLRIYAHVQTLLYNFFKYILIVYITYPIVQSFYDLWSSGVYSYIMPTLFWYPVPLEQSLFVYIVYLLFACFCSFCAGLIILSADLCLFSSVSQLMLHLDLLAQRIKELQPAEEGSLSALKAIIEYHQKILTIAKDVNSIFAPSILFSLASSSFILCFSAYQLLDDVSFIFALKVILLLGYEMKQVVITCYYGDKLMDSSANLFTAVYAHNWTDGSPVYKRLVLFMLVRTYRPIALKVAGISDVSLITLKQVLSTAYQIFTVLKTT

>BdorOr9 Cluster-8820.0_m.8086

MDFVQFFWFPNALYRIVGYDFQQLPRAHWRKALMKAFLIFTTISGICTRIYMLFQLRELILSGDILNSFRLGVYISYAIDSNVKFFVFLLNAKRLRVIYQSLSNEYPMTSMEQKLYQVDKYSFKRARIMIVSYLSVTNSILIGPMLQSIFMYIIDLFRYGYAAAAFSYLHPTPMSYNFNYCTPHYYILIYISEYLNGHFCTTTNLGTDLYVCTFAGQFCMQLEYLGSSLEAYEPSMDNSKADCKFLMEWIRKHQLMLDLCSELNEVFGTTLLFKLISNCAVFCIIVVQLKLEGFGFGFLNFLSFFFVTVAQFFMVCQYGQKLITISENLALCAYKNRWYNGSQTYKTLLFNIIARAQKPARLTAKGFQPISLATFQIVMTMTYRVFAVLQRALD

>BdorOr20 Cluster-23888.0_m.21800

MMPSFKSSEPAPTVPDFVDIPLFQIKFMGAKLFKWTPDEPRSKLQITLLGTFCVFATFNFTSMLLFVINDELATSLDITEFILFWGFALNAMMKGGTMVCFRRDIEFVLKGLVARHPKTEEEREAFQLVPYFRTINASNKYLSIWHLSITSIFALHPMVSSLLRYIWRDDTNESYDFTFPFMMAYYYDTNQPLTYAVSYFIQCCGAFYMSLLFLSGDLLLISMVQLVNMHFGYLIYKIESFQPTGTDADMRTLGPLLEYHNEILDYAERIDSTFSLATFLNYVGSCLVLCLIGLQIVLGSEALSVIKFIGFLVSTIVQVFFVSYFGNNLKDLSTGISDAFYNHPWYDANYKYMRMLVLPIARSQRYAHLTAFKFFEISMDSFKSLCTTSYQFFTLLRTSMEEEDS

>BdorOr8 Cluster-3774.0_m.3480

MISLSSKATISNTIASHNSYLTNHSYTHLKHTASKLQTILAPYRVLKEMLRCGEAVQPPHTCLFYFRSYIRLLGLWPAKRAAENQLYYFYNLLIMVLFSFFMATIICDLYEASSDFVLLGEDLVVVLGLYLIFFKMILFRMGNVDTDIIINEFDALHIKHARGLSGGPRNRRILQWQRSFFFGEMCFFSGFYILSLLLFAAMSLQPLLSQQTLPFRCKFPFGLNDPDEHPIAFVCVYFFQCFCTLYMLVAIVVMDSLGGNSFNQTTLNLRILCENIRHLGIVAAGASSSTSEAVAWRELREAVEFHQKIIGLMNRINQTFYWNYVSQMGASTFMICLTAFEALLAQDKPMVAMKFQTYMFSAFMQLLYWCWMGNRTYYDSMEVATAAYEIRAWYEHSPLLQRQLMFIIKRAQKPLEFRAKPLFGFTFASFTSILSTSYSYFALLRTMSD

>BdorOr25 Cluster-47806.0_m.43751

MKFSSELFNWSLTFMRRIGYFDQPRLVLLYLLSPISLSLMAYYRTYVIRHNFDEVIVNLFKISGSTMTIGRAFIVMCKSKKFLNFFESVDEWYQELHVRFAGGSSLFLFIYYLLTFLHFQREGDDVTLKKAHEYTKKIKKTSKTVLILTGITIFYIMFVQLLSTAGVGYKKLILDVAFPGVDFYESPLWEMMSILQGLWTAPIVIVSYVSYLCLTLIAIAFGIFLMKNLQSKLEGMNEMTDEEALKCIKKCVKDHVMIIKYHRDLEVLFSVNSFADVCIFAVIPCVIIIISTMDHDMSMLIGDIQLSIMVMISTFLVFWVGNNFCYENENIAKAAYNCNWENRNKEFRKYIPLIIITSQRPLQVFNDILQLFSYFNLFFITTMHKLLYINFFQLTAGGLKPINMEFFLTMVRCTYSLFTVLFTM

>BdorOr21 Cluster-27474.0_m.24937

RFLFCLIPIITYVGQIIHIFKSWNEDMGETSMNLHILLLKTHCLVRLWLMVKKPKDFERFFQCVEQWYRDIERNGDPQMVGALKEITKRTQLLSKMTIYVAAGGTIAAFFYPLSFDRRKHMITVQYPFVDALQTPFFEFLFLLQVLCLAPIILVLTLPFTNIYLISLMFGELVLKDLCVKLRNIRSENEETMLQEFKECIAYHRKIIDLCDDLQD

>BdorOr11 Cluster-17988.0_m.16383

MSPTPLSVPQLALAAVDTRSFLKLHWTCFKVLGINASNSSGYYLGYSVLLQVLVTFCYPLHLALALFSSADASTNIQNLAVCVVCVVCSAKFVIYATRMSRIRELESIIAALDARAQSPCERRYFVELRKEMRRITLGFLSIYAVVGVTAELMFFFRNEHNLLYPAWFPFDWRASDLKFYAAHSYQIVGISYQLLQNFVNDCLPTMALALLSAHIKLLGIRVSRIGYTTESPAANEEELLYCIKDQEQLYNMLNVIQNIISLPMFLQFTVTAINICLPLAALLFYVDAPFDRLYFVVYLVSLPLEIFPICYYGTTFQLLFNKLHVEMFFSNWVEQTHKFRKHMILFCERSLKSETATAGGIVRIHLDTFVSTCKAAYSLLAVIMKMNE
